# B‐cell lymphoma 2 family genes show a molecular pattern of spatiotemporal heterogeneity in gynaecologic and breast cancer

**DOI:** 10.1111/cpr.12826

**Published:** 2020-05-17

**Authors:** Jiajian Wang, Sidi Li, Shudai Lin, Shuying Fu, Li Qiu, Ke Ding, Keying Liang, Hongli Du

**Affiliations:** ^1^ School of Biology and Biological Engineering South China University of Technology Guangzhou China; ^2^ Department of Obstetrics and Gynecology Southwest Hospital Army Medical University (Third Military Medical University) Chongqing China

**Keywords:** BCL2 family, chromatin accessibility, long‐range gene regulation, molecular characteristic, pan‐cancer

## Abstract

**Objectives:**

BCL2 family proteins have been widely studied over the past decade due to their essential roles in apoptosis, oncogenesis and anti‐cancer therapy. However, the similarities and differences in the spatial pattern of the BCL2 gene family within the context of chromatin have not been well characterized. We sought to fill this knowledge gap by assessing correlations between gene alteration, gene expression, chromatin accessibility, and clinical outcomes in gynaecologic and breast cancer.

**Materials and methods:**

In this study, the molecular characteristics of the BCL2 gene family in gynaecologic cancer were systematically analysed by integrating multi‐omics datasets, including transcriptomics, chromatin accessibility, copy number variation, methylomics and clinical outcome.

**Results:**

We evaluated spatiotemporal associations between long‐range regulation peaks and tumour heterogeneity. Differential expression of the BCL2 family was coupled with widespread chromatin accessibility changes in gynaecologic cancer, accompanied by highly heterogeneous distal non‐coding accessibility surrounding the BCL2L1 gene loci. A relationship was also identified between gene expression, gene amplification, enhancer signatures, DNA methylation and overall patient survival. Prognostic analysis implied clinical correlations with *BAD*, *BIK* and *BAK1*. A shared protein regulatory network was established in which the co‐mutation signature of TP53 and PIK3CA was linked to the *BCL2L1* gene.

**Conclusions:**

Our results provide the first systematic identification of the molecular features of the BCL2 family under the spatial pattern of chromatin in gynaecologic and breast cancer. These findings broaden the therapeutic scope of the BCL2 family to the non‐coding region by including a significantly conserved distal region overlaying an enhancer.

## 1 INTRODUCTION

Each year, about 1 million new cases of gynaecologic malignant tumours are diagnosed and approximately 0.5 million women die as a result.^1^, ^2^ The formation and evolution of gynaecologic cancers are affected by many factors, including heredity, lifestyle, diet, exercise, sex and geographical environment.^1^ Gynaecologic and breast cancers, which are distinct classes of tumours, share some common characteristics, such their early formation from Mullerian ducts and their developmental control by female hormones.^3^ Recently, Berger et al^4^ identified uniform and unique characteristics of gynaecologic cancers at the molecular level; these molecular features provide a broad‐based, curated atlas for oncotherapy. However, our understanding of cell death and the mechanisms that cause tumorigenesis in gynaecologic tumours remains limited. Therefore, exploring common features of cell death regulation by apoptotic factors is important for developing new tumour therapies for gynaecologic cancers.

Over the last decade, cell cycle inhibitors, cell apoptosis inducers and cell proliferation inhibitors have been developed for oncotherapy of gynaecologic tumours. Targeting the apoptosis pathway is an important focus for the development of new treatments for malignant tumours.^5^ This especially includes the B‐cell lymphoma 2 (BCL2) family proteins, which are critical mediators of the apoptotic response and can be targeted by “BH3‐mimetic” small molecule compounds.^6^ Since the discovery of the BCL2 family in 1984,^7^ its members have expanded to include more than 15 that are involved in human cancer.^8^ The BCL2 family can be categorized into three groups: pro‐survival BCL2 family members; pro‐apoptotic multi‐BH‐domain members; and pro‐apoptotic “BH3‐only” members.^6^ Tumour cells frequently overexpress BCL2 family proteins to escape the apoptosis checkpoint.^8^ Copy number variations have been detected in the anti‐apoptotic members *MCL1* and *BCL2L1* across 26 human cancers, including gynaecologic cancers.^9^ Furthermore, overexpression of *MCL1* is thought to promote chemotherapy resistance and prolong cell survival in breast cancer and ovarian cancer.^10^, ^11^ However, loss of the representative pro‐apoptosis members *BAX*, *BIM* and *BBC3* is also a common trend in oncogenesis, facilitating tumour formation and progression through genomic deletion, silencing and mutation in several cancers.^12^, ^13^, ^14^ Despite the clear roles for these proteins, the correlation between BCL2 family members has not been systematically characterized using integrated multi‐omics data sets in gynaecologic cancers.

Previous research on the BCL2 family has been mostly concerned with overexpression or copy number variation and seldom with long‐distance regulation, and the distal regulation of the BCL2 family in gynaecologic cancers remains ambiguous.^6^, ^15^ Distal DNA regulatory elements can dramatically increase the expression of oncogenes/tumour suppressor genes during tumorigenesis.^16^, ^17^ However, recognizing and characterizing their contributions can be difficult. Because of polyA selection, relatively low depth genome sequencing and specific enhancer usage in rare cell populations, some enhancers have not been identified.^16^, ^17^ Recently, the assay of transposase‐accessible chromatin with sequencing (ATAC‐seq) method has proven useful for identifying functional spatiotemporal‐ and tissue‐specific distal regulation mechanisms between promoters and enhancers on a genomic scale.^18^ Distal regulatory regions of *BCL2* have been shown to target promoters based on chromatin accessibility dynamics in breast cancer.^19^, ^20^ However, potential enhancers of the BCL2 family are lacking in systematic research of gynaecologic cancer.

Apoptosis signalling pathways form a dynamic and complex network that maintains organismal homeostasis.^21^ Targeting BCL2 family proteins is an important approach in oncotherapy that strives to modulate the balance of apoptosis in order to control tumour cell death. Inhibitors of the BCL2 family (the BH3 mimetic drugs), such as ABT‐263 (Navitoclax) and ABT‐199 (Venetoclax), which target anti‐apoptotic members, have shown promising results in clinical trials of haematological cancers, but they have not yet been approved for clinical use for solid cancers, including gynaecologic cancer.^5^, ^22^, ^23^ Furthermore, BH3 mimetic drugs do not account for the great diversity in haematological and gynaecologic cancers, including the variable dependency on apoptotic proteins, and they vary in their safety and efficacy in the clinic. Suppression of the BCL2 family members may trigger disorganization of down/upstream signalling regardless of tumour heterogeneity, though the complex regulatory networks, especially the epigenetic signal and distal regulatory elements, are not well characterized.

To promote the development and application of drugs that target apoptosis signalling pathways, it is critical to further investigate molecular characteristics of the BCL2 family using large patient samples. Therefore, we evaluated differences in the patterns of genetic alteration by pro/anti‐apoptotic genes using a multi‐omics approach. Our strategy involved analysis of the transcriptome, copy number variation, the methylome and clinical outcomes. We also evaluated associations between methylation, gene amplification and chromatin accessibility, including spatiotemporal patterns and tumour heterogeneity. Finally, we identified genetic alterations in the BCL2 family network across gynaecologic cancers. An improved understanding of the common features and variations in the regulation patterns of the BCL2 family will be beneficial for selecting molecular targets and mechanisms to accelerate programmed cell death, leading to novel targeted drugs for treating gynaecologic cancer.

## 2 MATERIALS AND METHODS

### 2.1 Determination and comparison of genetic alterations

In this study, gynaecologic cancer mainly included breast invasive carcinoma (BRCA), high‐grade ovarian serous cystadenocarcinoma (OV), cervical squamous cell carcinoma and endocervical adenocarcinoma (CESC), uterine corpus endometrial carcinoma (UCEC) and uterine carcinosarcoma (UCS). Our analysis using cBioPortal (The cBio Cancer Genomics Portal) was performed in four stages: select data sets, select genomic profiles, define sample sets and type the interesting genes.^8^ Overall survival and BCL2 family gene alterations in pan‐cancers, including somatic mutations, amplification, deep deletion and DNA methylation, were measured through the cBioPortal database. We chose the “individual cancer study” query option within cBioPortal to explore genomic alterations for the following genes: *BCL2*, *BCL2L1*, *BCL2L2*, *MCL1*, *BAX*, *BAK1*, *BAD*, *BOK*, *BCL2L11*, *BMF*, *BID*, *NOXA*, *HRK*, *BBC3* and *BIK*. We selected the following data sets for downstream analysis: Breast Invasive Carcinoma (TCGA (Nature 2012), n = 463), Ovarian Serous Cystadenocarcinoma (TCGA (Nature 2011), n = 316), Cervical Squamous Cell Carcinoma and Endocervical Adenocarcinoma (TCGA (Provisional), n = 190), Uterine Corpus Endometrial Carcinoma (TCGA (Nature 2013), n = 232) and Uterine Carcinosarcoma (TCGA (PanCancer Atlas), n = 56).

### 2.2 Exploring gene expression levels between normal tissues and tumour samples

Gene Expression Profiling Interactive Analysis (GEPIA) is a free and interactive online database for cancer mRNA data, which consists of 9736 tumours and 8587 normal samples from The Cancer Genome Atlas (TCGA) and Genotype‐tissue Expression dataset projects (GTEx).^24^ The expression levels of BCL2 family genes in gynaecologic tissues and normal samples were measured using the Boxplot module in GEPIA.^25^

### 2.3 Evaluating chromatin accessibility of the BCL2 gene family

The assay of transposase‐accessible chromatin with sequencing (ATAC‐seq) can sensitively map chromatin accessibility of active genes and also can identify functional spatiotemporal and tissue‐specific regulatory mechanisms between promoters and distal sites within a 500 kb genomic region surrounding the transcriptional start site (TSS).^26^ To explore and affirm the chromatin state of BCL2 family gene loci in gynaecologic cancer, we compared differential chromatin‐accessibility (ATAC‐seq) data sets from the UCSC Xena browser with published histone modification ChIP‐seq datasets from gynaecologic tumour samples, including EP300, TEAD4, H3K4me1 and H3K27ac (Table S1). To highlight both differences and similarities between BCL2 family gene characteristics in gynaecologic cancers, we selected male‐specific tumour testicular germ cell tumours as a non‐gynaecologic cancer. The published histone modification data sets were collected in public databases including Gene Expression Omnibus (GEO), Encyclopedia of DNA Elements (ENCODE), and the Roadmap Epigenetics Project. The analysis of ChIP‐seq data sets was divided into three parts: mapping the reads, calling the peaks and annotating the peaks. The raw data of SRA files that were downloaded from GEO (ftp://ftp‐trace.ncbi.nih.gov/sra/srainstant/reads/ByRun/sra/SRR/) used fastq‐dump (https://ncbi.github.io/sra‐tools/fastq‐dump.html) to convert SRA files to FASTQ files. First, we mapped these FASTQ files to the human genome (hg38) using BWA with the BWA‐MEM algorithm.^27^ Then, we used MACS2 to call the statistically significant peaks with the macs2 callpeak command.^28^, ^29^ Finally, we annotated the open‐/closed peaks, including average conservation regions using HOMER with annotatePeaks.pl module.^30^ The distal chromatin accessibility of the BCL2 family in gynaecologic cancer was analysed and visualized with the UCSC Xena Browser and the Integrative Genomics Viewer.^31^

### 2.4 Network analysis of the BCL2 family

To explore the BCL2 family signalling networks in gynaecologic cancer, we queried the BCL2 gene in cBioPortal.^32^ The establishment of the pathway and interaction in cBioPortal was mainly based on the Human Reference Protein Database (HPRD), Reactome, National Cancer Institute (NCI) and the Memorial Sloan‐Kettering Cancer Center (MSKCC) Cancer Cell Map. To provide a well‐calibrated regulatory network, each member with a ≥30% alteration frequency was retained according to an established workflow with modifications in the BCL2 family regulation network.^32^

### 2.5 Statistical analysis

Statistical analyses of mRNA expression levels were performed in GEPIA. Differences between two groups were compared using t test, and differences between multiple groups were compared using one‐way ANOVA. Family genes with *P* values <0.01 are considered significantly differentially expressed genes. Pearson/Spearman tests and linear regression equations were employed to investigate the correlation between DNA methylation and mRNA expression. If the Pearson/Spearman coefficient is <0, it indicates that DNA methylation and mRNA expression are negatively correlated. Linear regression equations can be utilized to show a gradual downward tendency. Kaplan‐Meier curves were prepared to evaluate overall survival, and the log‐rank test was used to calculate *P* values. *P* < .05 was regarded as statistically significant in Kaplan‐Meier survival curve analyses.

## 3 RESULTS

### 3.1 Gene alteration of the BCL2 family in pan‐cancer

**FIGURE 1:**
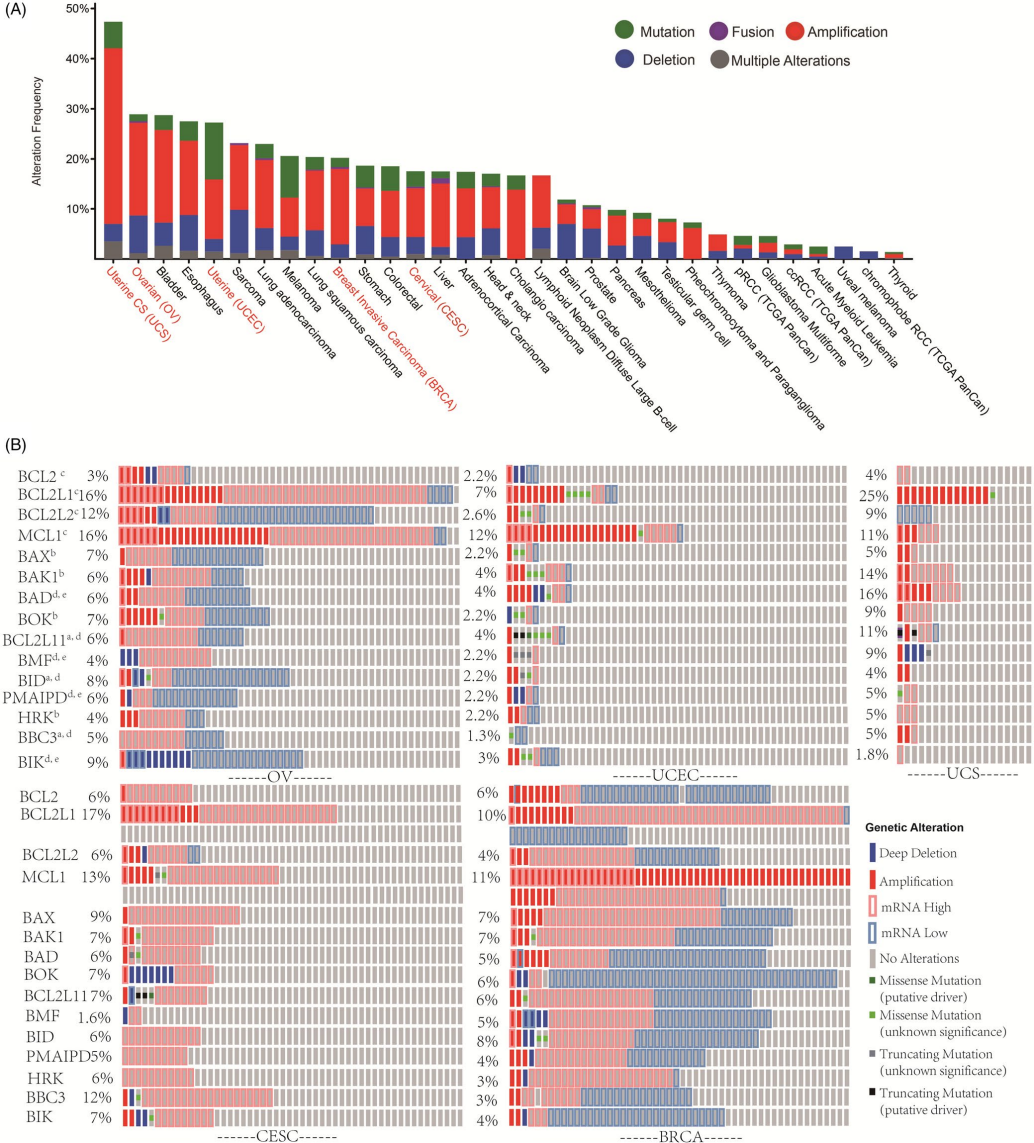
Gene alteration of the BCL2 family in pan‐cancer. A, The alteration frequencies of the BCL2 family across the TCGA PanCancer atlas. The horizontal axis represents the types of cancer, and the vertical axis represents the alteration frequencies of the BCL2 family. Green coding indicates non‐synonymous mutations, purple coding indicates gene fusions, red coding indicates gene amplification, blue coding indicates deep deletions and grey coding indicates multiple alterations. Gynaecologic cancers are marked in red font. B, Genetic alteration of the BCL2 family in gynaecologic cancer. The functional classification of the BCL family member is indicated: ^a^Activators; ^b^Effectors; ^c^Guardians; ^d^Initiators; ^e^Sensitizers

BCL2 family proteins have been referred to by different names in different studies. To facilitate our study of the BCL2 family, we adopted a unified terminology with gene names from NCBI (Table S2). The fifteen members of the BCL2 family that are expressed in gynaecologic cancers (*BCL2*, *BCL2L1*, BCL2L2, *MCL1*, *BAX*, *BAK1*, *BAD*, *BOK*, *BCL2L11*, *BMF*, *BID*, *NOXA*, *HRK*, *BBC3* and *BIK)* present aberrant expression in many cancers and may be associated with chromosomal translocations, gene amplification, upregulated gene transcription, altered post‐translational processing and tumour progression.^6^, ^8^, ^9^ Therefore, we surveyed their genomic alterations in TCGA pan‐cancer datasets. The gene alteration frequencies of the BCL2 family, including mutations, deletions and amplifications, are shown (Figure 1A; Tables S3). The data show that gene alteration frequencies in the BCL2 family were more widely found in gynaecologic cancer than many other cancers. In general, BCL2 family amplification was more frequent than deep deletion and missense mutations across pan‐cancer. This was particularly notable for gynaecologic cancers, including UCS (35.09%), OV (18.46%) and UCEC (11.91%). More than 20% of cases in these data sets had BCL2 family amplification. Given the roles of BCL2 family proteins in regulating apoptosis, these results suggest that they are integrally involved in gynaecologic cancer.

To further explore the genetic alteration of the BCL2 family in gynaecologic cancer, we generated an Oncoprint of their alteration frequencies using cBioPortal. UCS, CESC, OV, BRCA and UCEC showed 8.92%,7.71%, 7.67%, 5.93% and 3.55% average alteration rates, respectively (Figure 1B). Interestingly, we observed low mRNA expression in some patient samples of OV and BRCA, especially for the pro‐apoptotic member. On the contrary, the alterations in the anti‐apoptotic members, including *BCL2L1* and *MCL1,* predominantly included mRNA upregulation and gene amplification. Consistently, driver mutations and downregulation of mRNA seldom were observed in gynaecologic patient samples for *BCL2L1* and *MCL1*. These results suggest that for the gynaecologic cancers, anti‐apoptosis BCL2 family members tended to be upregulated rather than downregulated, with gene amplification at higher frequency for the anti‐apoptosis compared with the pro‐apoptosis members.

### 3.2 The mRNA expression of the BCL2 family is coupled with widespread chromatin accessibility changes in gynaecologic cancer

**FIGURE 2:**
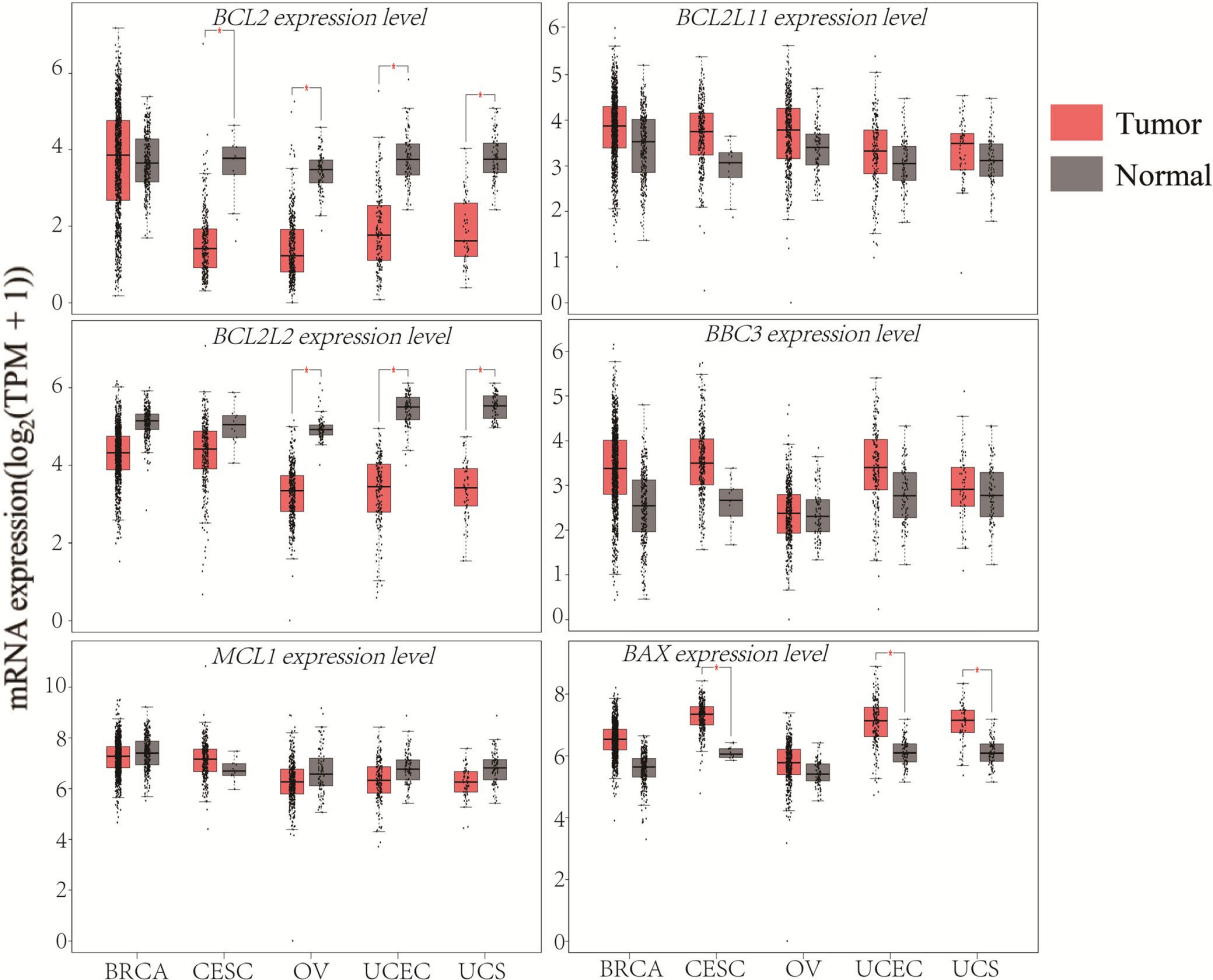
The mRNA expression patterns for pro/anti‐apoptotic BCL2 family members in gynaecologic cancer. The anti‐apoptotic members *BCL2*, *BCL2L2*, and *MCL1* were downregulated, whereas the pro‐apoptotic members *BCL2L11*, *BBC3* and *BAX* were upregulated in gynaecologic cancer compared with normal tissues (N (Tumour) = 1085 and N (Normal) = 291 for BRCA, N (Tumour) = 306 and N (Normal) = 13 for CESC, N (Tumour) = 426 and N (Normal) = 88 for OV, N (Tumour) = 174 and N (Normal) = 91 for UCEC, N (Tumour) = 57 and N (Normal) = 78 for UCS). The red box represents tumour samples, the black box represents normal samples. The red star indicates statistical significance (*P* < .01)

To assess the overall consequence of BCL2 family upregulation on chromatin accessibility, we first evaluated the expression changes in gynaecologic cancer using TCGA and GTEx databases. The different patterns of mRNA expression between the pro/anti‐apoptotic members in gynaecologic cancer are shown in Figure 2 and Figure S1. In general, the pro‐apoptotic members of the BCL2 family, including *BAX*, *BAK1*, *BAD*, *BCL2L11*, *BMF*, *BID*, *NOXA*, *HRK*, *BBC3*, *BIK,* were upregulated in gynaecologic cancer. The anti‐apoptotic members *BCL2* and *BCL2L2* were downregulated in gynaecologic cancer; however, other anti‐apoptotic members *MCL1* in BRCA and *BCL2L1* showed upregulation in gynaecologic cancer. Therefore, there was a unique pattern of regulation of BCL2 family gene expression for the pro/anti‐apoptotic members, with the pro‐apoptotic members more highly expressed in gynaecologic cancer compared to normal tissues.

**FIGURE 3:**
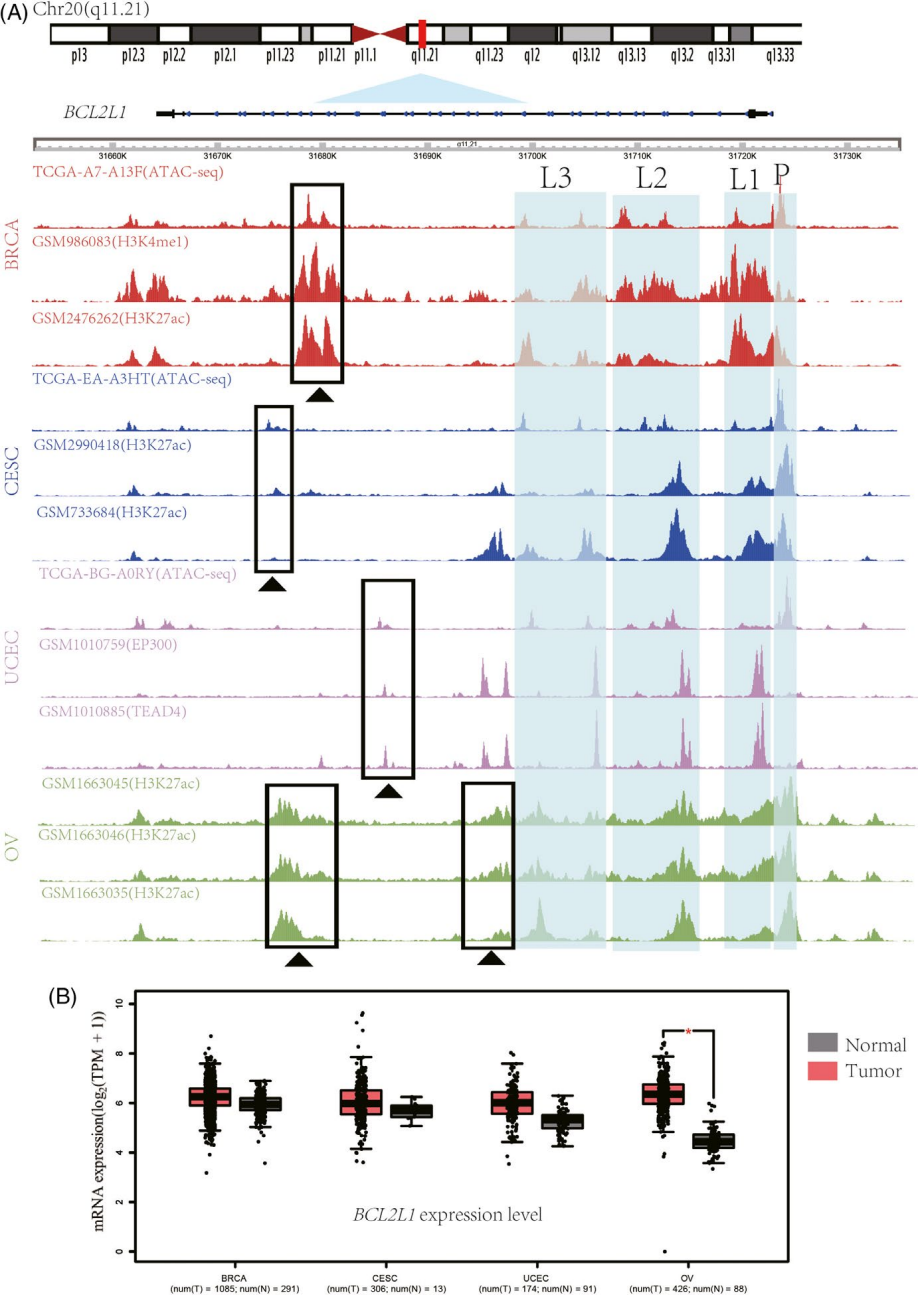
The mRNA expression at the BCL2L1 locus in gynaecologic cancers is coupled with widespread chromatin accessibility changes. A, Normalized open chromatin tracks in the *BCL2L1* gene locus in gynaecologic cancer. Three inferred peaks at promoters are indicated (L1 to L3, positioned at chr20: 31713198‐31714227, chr20: 31716244‐31717493 and chr20: 31720069‐31720570). Highly variable distal regulatory landscapes are marked with black triangles. The regions range from chr20:31,654,911 to 31,734,116. B, Upregulated *BCL2L1* mRNA expression in gynaecologic cancer compared with normal tissues (N (Tumour) = 1085 and N (Normal) = 291 for BRCA, N (Tumour) = 306 and N (Normal) = 13 for CESC, N (Tumour) = 174 and N (Normal) = 91 for UCEC, N (Tumour) = 426 and N (Normal) = 88 for OV). The red star indicates the p‐value threshold (*P* < .01)

The presence of chromatin accessibility changes accompanied by H3K4me1 or H3K27ac histone modification outside promoter regions is indicative of an active enhancer.^33^, ^34^, ^35^, ^36^, ^37^ Therefore, we explored whether the links between distal regulatory elements and promoters were specifically correlated with histone modifications. To this end, we used ATAC‐seq, which accurately identifies tissue‐ or stage‐specific distal links regardless of the number of samples. We compared differential chromatin‐accessibility ATAC‐seq datasets from the UCSC Xena browser with published histone modification ChIP‐seq datasets from gynaecologic tumour samples, and found that neither open‐peaks nor closed‐peaks were enriched in histone modifications. However, there was overlap between open regions and functionally verified enhancers at BCL2 family gene loci as modelled by the *BCL2L1* locus (Figure 3 and Figure S2). The open peaks of the BCL2 family in gynaecologic cancer exhibited a wide genomic distribution that overlapped the promoters, intergenic regions and introns (Figures [Link], [Link], [Link], [Link], [Link], [Link], [Link], [Link], [Link]). As expected, active signatures of chromatin accessibility were observed near the TSS. These observations support the positive correlation between open chromatin and gene expression. There were also distal active peaks of BCL2 family genes that were relative well conserved across different gynaecologic cancer types. Overall, two thirds (10/15; *BCL2*, *BCL2L1*, *BAD*, *BAK1*, *BID*, *BIK*, *BMF*, *BOK*, *HRK* and *PMAIP1*) of the BCL2 family members presented distal connections in which open chromatin sites were not uniformly distributed around or within the gene body. Importantly, these chromatin accessibility signatures in the BCL2 family presented various spatiotemporal patterns. We found that these non‐coding distal peaks were clustered in the region surrounding the BCL2 family genes: downstream from the TSS (at distances ranging from 0.6 to 200 kb), including introns; and upstream of the TSS (at distances ranging from 1.5 to 370.4 kb). The open peaks around the promoter region of *BCL2L1* gene locus showed ubiquitous accessibility; however, the distal regulatory region of the *BCL2L1* gene locus showed variable accessibility across gynaecologic and breast cancers. These data suggest that the regulation of *BCL2L1* open peaks in the promoter differs from that in the distal regulatory region, which varies according to the type of cancer and could be an important consideration in drug design. To our knowledge, this study provides the first systematic research on the long‐range peak‐promoter communication of the BCL2 family.

### 3.3 The relationship between promoter DNA methylation and BCL2 gene expression in gynaecologic cancer

**FIGURE 4:**
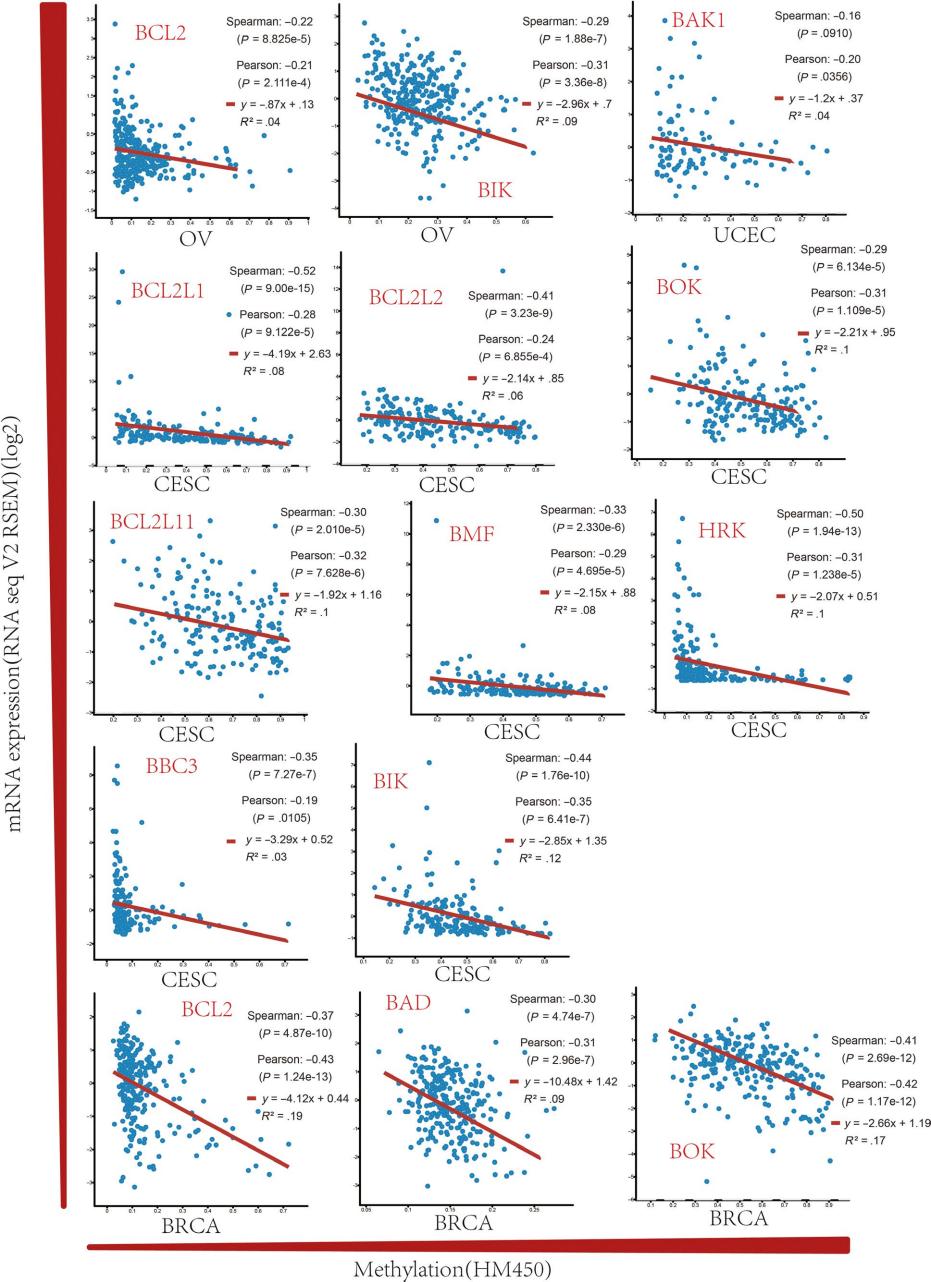
The promoter methylation levels of the BCL2 family in gynaecologic cancer. A negative correlation exists between mRNA expression and promoter DNA methylation for *BAD* and *BOK* in BRCA; *BOK* in CESC; *BRCA* and *BAK1* in UCEC; *BIK* in OV and CESC; *BCL2* in OV and BRCA; and *BCL2L1*, *BCL2L2*, *BCL2L11*, *BMF*, *HRK* and *BBC3* in CESC

To further investigate the regulatory mechanism of BCL2 family expression in gynaecologic cancer, we examined the correlation between promoter DNA methylation and mRNA expression through Genetic Profile analysis in cBioportal. A significant negative correlation was observed between mRNA expression and promoter DNA methylation for *BAD* and *BOK* in BRCA and CESC. A negative correlation was also observed for *BAK1* in UCEC, *BIK* in OV and CESC, *BCL2* in OV and BRCA, and *BCL2L1*, *BCL2L2*, *BCL2L11*, *BMF*, *HRK* and *BBC3* in CESC (Figure 4). The results demonstrate that the dysregulation of BCL2 family expression in gynaecologic cancer is associated with promoter DNA methylation.

### 3.4 Prognostic analysis of selected genes with the enhancer signature in gynaecologic cancer

**FIGURE 5:**
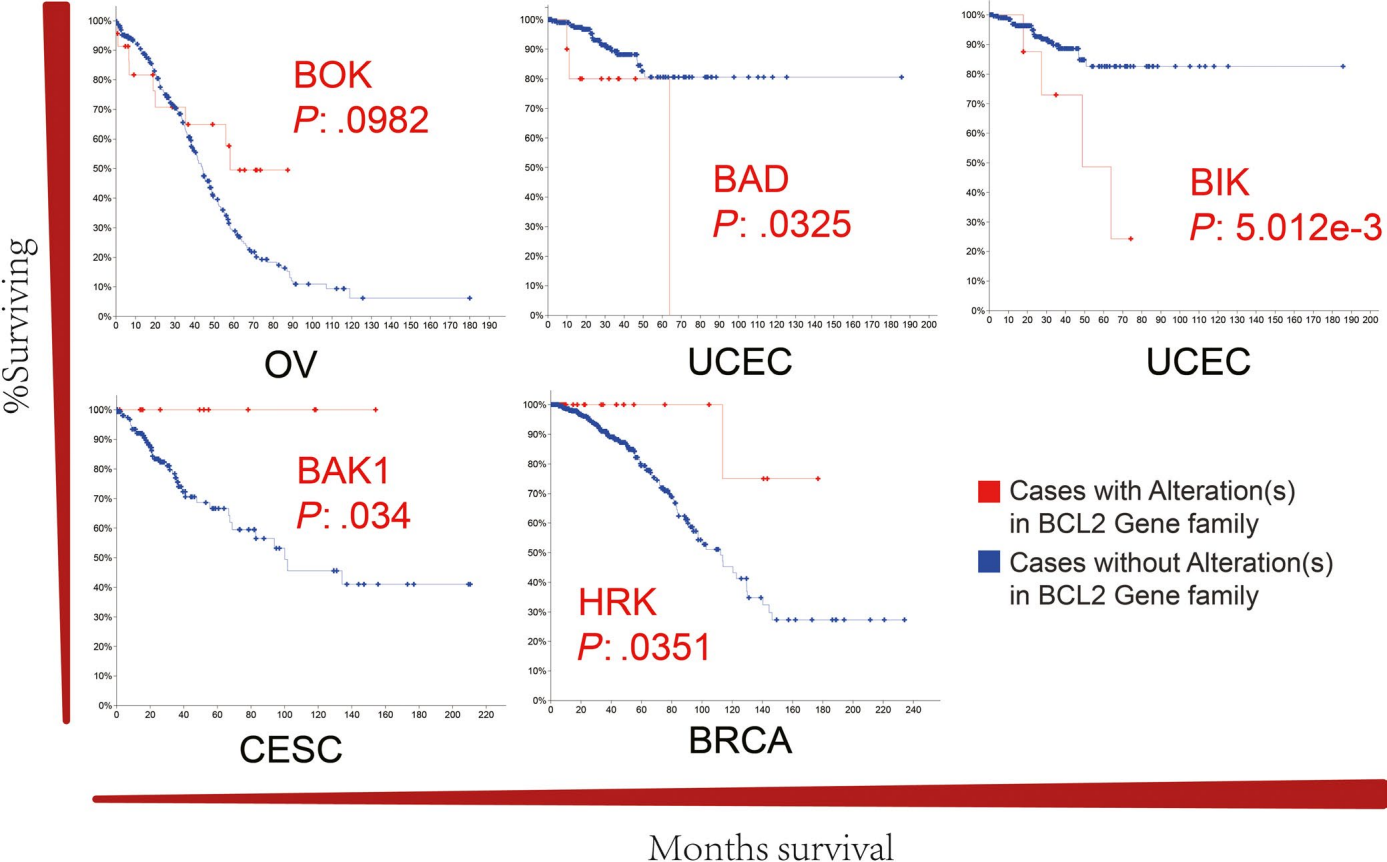
The overall survival in gynaecologic cancer with BCL2 family alteration. Alterations in *BOK* in OV, *BAD* and *BIK* in UCEC, *BAK1* in CESC, and *HRK* in BRCA were significantly associated with overall survival

To explore the effect of BCL2 family alteration on patient survival in gynaecologic cancer, we compared the overall survival for cases with and without gene alteration (Figure 5). Kaplan‐Meier analysis indicated that gene alteration in *BOK* was significantly associated with favourable prognosis in OV (*P* < .1), which is consistent with the results of the genetic alteration analysis (Figure 1). Similarly, favourable prognosis was found for *BAK1* in CESC (*P* < .05) and *HRK* in BRCA (*P* < .05). Nevertheless, gene alterations in *BAD* and *BIK* were associated with poor survival in UCEC (*BAD*, *P* < .05; *BIK*, *P* < .01). These results are supported by the high expression of *BAK1* in CESC and *BAD* and *BIK* in UCEC (Figure 2). However, no correlation between mRNA expression and survival was observed for *BCL2L1*.

### 3.5 The regulatory network of the BCL2 family in gynaecologic cancer

**FIGURE 6:**
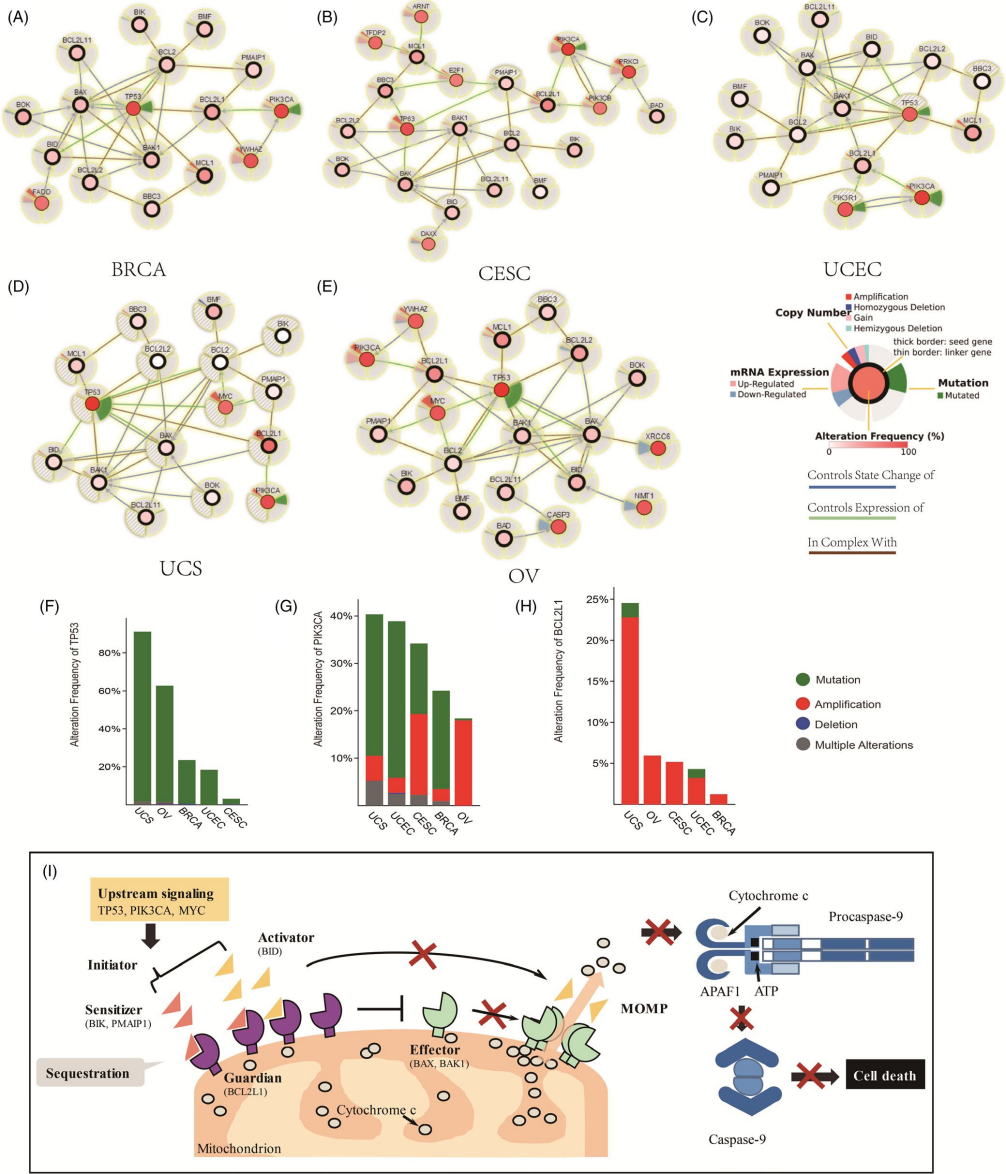
The regulatory network of the BCL2 family in gynaecologic and breast cancer. A‐E, The regulatory network of the BCL2 family members with 30% alteration frequency. The networks from cBioportal analysis show alteration frequency, mRNA expression, copy number and mutation coding by various colours in different circular areas across gynaecologic cancers. F‐H, The co‐mutation/amplification signatures for *TP53*, *PIK3CA* and *BCL2L1*, for which gene mutation or amplification/upregulation was found more frequently. I, The BCL2 family‐mediated mitochondrial apoptosis pathway in gynaecologic and breast cancer. In the death signal pathway, upstream signalling unleashes initiator proteins, including sensitizers (BIK, PMAIP1, etc) and activators (BID, etc). Subsequently, these proteins are bound and immediately sequestered by guardian proteins (including BCL2L1). Because the transcriptional expression of the *BCL2L1* gene tends to have more amplification events and chromatin accessibility, BCL2L1 protein never reaches a state of saturation. Guardian proteins prevent effector proteins from oligomerizing and causing mitochondrial outer membrane permeabilization (MOMP), which prevents cytochrome c release from the mitochondria. Without cytochrome c, APAF1 cannot dimerize with procaspase‐9 to form the apoptosome. Thus, guardian proteins mediate the immortalization of cells

The dynamic regulatory network of the BCL2 family protein suggests the importance of mitochondrial outer membrane permeabilization (MOMP), but the mechanisms behind the interactions remain controversial.^15^, ^21^, ^38^ To identify the pattern of BCL2 family protein inhibition of MOMP, we comprehensively inferred the pan‐cancer BCL2 family interaction signal network in gynaecologic cancer (Figure 6A‐E). The resulting networks from cBioportal analysis were highly structured across gynaecologic cancer types and were dominated by genetic alteration in BCL2 family members, including *BCL2*, *BCL2L1*, *BAK1* and *BAX*. Furthermore, transformation‐related protein 53 (*TP53*), transformation‐related protein 63 (*TP63*), phosphatidylinositol‐4,5‐bisphosphate 3‐kinase catalytic subunit alpha (*PIK3CA*) and Myc‐related translation/localization regulatory factor (*MYC*) were found to have frequent gene mutation or amplification/upregulation (Figure 6F‐H). These interactions are consistent with the previously pattern of apoptotic switch that bridges the connection between initiators, effectors and guardians^5^, ^21^ (Figure 6I). Importantly, we observed that both *TP53* and *PIK3CA* had high‐frequency mutations (Figure 6F‐G), and simultaneously were significantly associated with genes for activators (BID), effectors (BAX and BAK1) and guardians (MCL1, BCL2 and BCL2L1) in BRCA, UCEC, UCS and OV. Unexpectedly, transformation‐related protein 63 (TP63), rather than TP53, was predicted to regulate the sensitizers (PMAIP1), activator (BBC3) and effectors (BAX) in CESC (Figure 6B). Fas‐associated via death domain (FADD) and tyrosine 3‐monooxygenase/tryptophan 5‐monooxygenase activation protein zeta (YWHAZ) were also highly specific connected in the BRCA network, while both phosphatidylinositol‐4,5‐bisphosphate 3‐kinase catalytic subunit beta (*PIK3CB*) mutation in CESC, and phosphoinositide‐3‐kinase regulatory subunit 1 (*PIK3R1*) mutation in UCEC controlled the expression of *BCL2L1*. X‐ray repair cross‐complementing 6 (*XRCC6*), N‐myristoyltransferase 1 (*NMT1*) and caspase 3 (*CASP3*) in OV showed specific downregulated expression. Furthermore, significant gene amplification of *BCL2L1* and *MYC* was observed in UCS. Therefore, the BCL2 family protein network shows common features of co‐mutations in *TP53* and *PIK3CA* but varies in other genes for different types of gynaecologic cancer.

## 4 DISCUSSION

Apoptosis is a continuous programmed cell death event that is responsible for periodic control of damaged cells during normal development, maintaining a balance of organismal homeostasis and preventing pathological autoimmunity and tumorigenesis.^6^ Unfortunately, apoptosis inhibition or escape in tumour cells leads to abnormal survival and accumulation of dysfunctional cells.^8^ Apoptosis is controlled by the BCL2 protein family, which includes pro‐apoptotic and pro‐survival members. Some members of the family, such as BCL2, are overexpressed in tumour cells, thus breaking the balance between life and death and resulting in unlimited proliferation^8^, ^19^ (Figure 6I). Furthermore, the regulatory relationships among these proteins and DNA amplification, mutation, methylation, overall survival and chromatin accessibility have not been comprehensively examined in gynaecologic cancers.

Here, we evaluated the molecular characteristics of BCL2 family genes under the spatial pattern of chromatin in gynaecologic cancers. As noted, most prior studies have identified initiators, effectors and guardians of the BCL2 family within a unified dynamic model in a variety of cancers, leading to anti‐apoptosis and drug resistance in tumour cells^21^ (Figure 6I). Recent systematic analyses have uncovered the dynamics of BCL2 family regulation, particularly for anti‐apoptotic members, as the most frequent somatic copy‐number alteration in human cancer.^9^, ^15^, ^21^ We found that *BCL2L1* and *MCL1* show higher expression levels as compared to other BCL2 members, which is consistent with the genetic alteration results. Our observation that anti‐apoptotic members are not widely expressed in gynaecologic cancers agrees with previous studies, and low expression level and deep deletion was also observed in some patient samples for anti‐apoptotic members.^39^, ^40^ In terms of distal non‐coding chromatin accessibility, both *BCL2* and *BCL2L1* have long‐range promoter interactions that affect gene expression. Conversely, *BCL2*, *BCL2L2*, and *MCL1* show downregulation across 2‐3 different cancer types, despite distal *BCL2* regulation.^41^ These phenomena may be due to the enforced expression or activation of miRNAs that normally suppress BCL2 family expression, such as miR‐15a or miR‐16.1 targeting of *BCL2*; miR‐29, miR‐125 and miR‐193 targeting of *MCL1*; or let‐7 targeting of *BCL2L2*.^41^, ^42^, ^43^, ^44^ Of the remaining overexpressed members, *BAX*, *NOXA*, *BIK*, *BID* and *BAK* are pro‐apoptotic. Enhancer signatures can be detected, and the mRNA expression levels of these pro‐apoptotic genes may be affected by these distal signatures. However, active effectors (*BAX* and *BAK*) or initiator (*PMAIP1*, *BIK* and *BID*) cannot lead to apoptosis in tumours, and these pro‐apoptotic proteins may be sequestered by guardians (*BCL2* and *BCL2L1*) that have more potent enhancer activity to propel the anti‐apoptotic mechanisms.

In our study of overall survival, *BOK* in OV, *BAD* and *BIK* in UCEC, *BAK1* in CESC, and *HRK* in BRCA show noteworthy associations with overall survival. BAD and BIK regulate tumour growth in many cancers.^45^, ^46^, ^47^, ^48^ Furthermore, worse overall survival may be triggered by activated guardians (the antiapoptotic proteins eg BCL2L1), which can bind and neutralize BAD and BIK to mediate the inhibition of apoptosis.^15^, ^21^, ^38^ BAD phosphorylation has recently been reported to promote tumour cell survival, and post‐translational modification might therefore contribute to impaired pro‐apoptotic proteins.^49^ Overexpression of BAK1 has previously been associated with a favourable prognosis in breast cancer,^50^ while BOK, BAK1 and HRK as effectors are inserted into the mitochondrial outer membrane, resulting in MOMP without sequestration by the guardians.^15^, ^21^, ^38^ Overall, our results imply that *BAD*, *BIK* and *BAK1* may be prognostic genes for clinical effects in gynaecologic cancer.

It is noteworthy that deep deletion and mutation were not observed widely in gynaecologic cancers, especially for pro‐apoptotic genes, which differs from previous studies.^6^, ^8^ For example, in humans, multiple tumours mutations and deletions occur at higher frequency in *BAX* and *BAK1*,^14^, ^51^, ^52^, ^53^ but mRNA upregulation serves as the main mechanism of *BAX* and *BAK1* activation in gynaecologic cancer. The alteration of pro‐apoptotic genes indicates that dysregulated mechanisms may be influenced by epigenetic or distal enhancer‐promoter contacts to control gene expression in gynaecologic cancer. Our results suggest that anti‐apoptotic genes and pro‐apoptotic genes display different mechanisms of dysregulation in gynaecologic cancer.

DNA methylation in promoter regions can be highly heterogeneous during tumorigenesis and progression,^54^ and thus we explored the relationship between DNA methylation and BCL2 family expression level in gynaecologic cancers. In general, almost all methylation of BCL2 family genes obviously influenced gene expression. The data suggest that *BCL2L2, BCL2L11* and *BBC3* might be affected by DNA methylation and gene amplification. Furthermore, *BCL2*, *BCL2L1*, *BAK1*, *BAD*, *BOK*, *BIK*, *BMF* and *HRK* might be more influenced by gene amplification and distal regulatory elements, with far less exposure than other members to methylation modification.

Beyond a limited number of genetic differences, the network from cBioportal analysis was highly structurally similar for different gynaecologic cancers. Our discovery of co‐mutation signatures for *TP53* and *PIK3CA* with *BCL2L1* is novel and was not revealed in a previous study of gynaecologic cancer.^55^, ^56^ Co‐mutation of TP53 and PIK3CA is a primary mediator of anti‐apoptotic protein inhibition of MOMP that lead to a more aggressive phenotype with a worse prognosis in breast cancer.^56^ These proteins are connected by co‐mutation signatures; *MCL1* and *BCL2L1* transcriptional activity are consistent with chromatin accessibility of the region surrounding the gene loci with more than one predicted distal enhancers. Therefore, we hypothesize that the co‐mutation signature of *TP53* and *PIK3CA* acts to stimulate the formation of chromatin loops of *BCL2L1* gene loci to strengthen its transcriptional activity. BCL2L1, as the only upregulated anti‐apoptotic member, prevents the activation and oligomerization of pro‐apoptotic members that act as guardians against the effectors or activators on the mitochondrial membrane, which maintains the balance of mitochondrial membrane potential and thus prevents the release of cytochrome c to suppress caspases.^39^, ^57^, ^58^, ^59^ Using *BCL2L1*, *TP53/TP63* and *PIK3CA* as possible potential prognosis markers may therefore be an effective approach for diagnosis and treatment of gynaecologic cancer.

The resulting network models showed specific differences in *YWHAZ* amplification in BRCA, *PIK3CB* amplification in CESC, *PIK3R1* mutation in UCEC, and downregulation of *XRCC6*, *NMT1* and *CASP3* in OV, which suggests that the BCL2 family protein network can be used to identify different types of gynaecologic cancer. YWHAZ has been shown to stimulate lung cancer cell proliferation and metastasis and promote the invasion of breast cancer cells,^60^ suggesting that it might serve as a therapeutic target of breast cancer.^61^ Mutation of PIK3CB, as the catalytic subunit in the PI3K signalling pathway, drives tumour cell growth and migration.^62^ PIK3CB has been reported as a selective survival factor in glioblastoma.^63^ Furthermore, co‐mutation of PIK3R1 and PIK3CA is associated with oncogenesis and hyperactivity of the PI3K signal pathway in breast cancer, supporting an oncogenic role of the co‐mutation pair.^64^ Loss of PIK3R1 is an effective therapeutic mechanism for PIK3CA‐positive breast cancers.^65^ On the other hand, activation of CASP3 is involved in the initiation of cell apoptosis,^66^ inhibition of NMT1 regulates breast cancer oncogenesis by the JNK pathway,^67^ and inactive XRCC6 fails to protect genomic integrity.^68^ Therefore, our findings further validate previous studies demonstrating that downregulation of XRCC6, NMT1 and CASP3 is significantly associated with tumorigenesis.

Long‐range enhancer‐promoter gene expression is facilitated and constrained by the 3D architecture of mammalian genomes, which plays a key role in disease.^69^ We demonstrated that the significant differential expression of the BCL2 family shows a signature of chromatin accessibility. We systematically identified spatiotemporal patterns of gene expression of the BCL2 family orchestrated by distal chromatin accessibility. The chromatin accessibility profile had a similar distribution in different tumour samples, which is likely dictated by the folding of chromatin loops within the 3D topography of the genome to bring enhancers in close spatial proximity with promoters and accelerate RNA polymerase recruitment.^69^, ^70^, ^71^, ^72^ These findings are significant for designing new medicines based on the molecular characteristics of high tumour heterogeneity surrounding the *BCL2L1* gene loci, including those that target specific topological features.

In conclusion, as the first systematic analysis of molecular feature of the BCL2 family under the spatial pattern of chromatin in gynaecologic cancer, our study broadens the therapeutic scope of the BCL2 family to the distal non‐coding region. We demonstrated that differential expression of BCL2 family members occurs at different frequencies. Furthermore, we identified the relationship between overall survival, enhancer signature, gene amplification and DNA methylation. Our results also establish a shared protein regulatory network in which the co‐mutation signatures of *TP53* and *PIK3CA* interact with *BCL2L1*, which provides a new strategy for biomarker identification in oncotherapy.

## CONFLICT OF INTEREST

All authors declare that they have on conflict of interest.

## AUTHOR CONTRIBUTIONS

Jiajian Wang and Hongli Du planned the study. Jiajian Wang and Sidi Li performed the gene expression and chromatin accessibility of BCL2 family, generated the results of DNA methylation and overall survival, constructed the regulatory network, designed the analytical pipeline, analysed and interpreted the data, and wrote the manuscript with help from Sidi Li and Hongli Du. Sidi Li imaged and analyzed network characteristics. Shudai Lin and Shuying Fu led revisions to the manuscript. Sidi Li, Li Qiu, Ke Ding, and Keying Liang provided outstanding guidance with the proposed model of *BCL2L1* and the mitochondrial apoptosis pathway. All authors discussed the results and commented on the manuscript.

## Supporting information

Fig S1

Fig S2

Fig S3

Fig S4

Fig S5

Fig S6

Fig S7

Fig S8

Fig S9

Fig S10

Table S1‐S3

## Data Availability

The data that support the findings of this study are available in cBioportal database, GEPIA database, UCSC Xena browser, GSM986083, GSM2476262, GSM2990418, GSM733684, GSM1010759, GSM1010885, GSM1663045, GSM1663046 and GSM1663035. These data were derived from the following resources available in the public domain: https://www.cbioportal.org/, http://gepia.cancer‐pku.cn/, https://xenabrowser.net/datapages/?hub=https://atacseq.xenahubs.net:443, https://gdc.cancer.gov/about‐data/publications/ATACseq‐AWG and https://www.ncbi.nlm.nih.gov/gds/.

## References

[1] Siegel RL , Miller KD , Jemal A . Cancer statistics, 2019. CA Cancer J Clin. 2019;69(1):7‐34. 30620402 10.3322/caac.21551

[2] Siegel RL , Miller KD , Jemal A . Cancer statistics, 2015. CA Cancer J Clin. 2015;65(1):5‐29. 25559415 10.3322/caac.21254

[3] Mullen RD , Behringer RR . Molecular genetics of mullerian duct formation, regression and differentiation. Sex Dev. 2014;8(5):281‐296. 25033758 10.1159/000364935 PMC4378544

[4] Berger AC , Korkut A , Kanchi RS , et al. A comprehensive pan‐cancer molecular study of gynecologic and breast cancers. Cancer Cell. 2018;33(4):690‐705. 29622464 10.1016/j.ccell.2018.03.014 PMC5959730

[5] Czabotar PE , Lessene G , Strasser A , et al. Control of apoptosis by the BCL‐2 protein family: implications for physiology and therapy. Nat Rev Mol Cell Bio. 2014;15(1):49‐63. 24355989 10.1038/nrm3722

[6] Delbridge ARD , Grabow S , Strasser A , et al. Thirty years of BCL‐2: translating cell death discoveries into novel cancer therapies. Nat Rev Cancer. 2016;16(2):99‐109. 26822577 10.1038/nrc.2015.17

[7] Tsujimoto Y , Finger LR , Yunis J , et al. Cloning of the chromosome breakpoint of neoplastic B cells with the t(14;18) chromosome translocation. Science. 1984;226(4678):1097‐1099. 6093263 10.1126/science.6093263

[8] Hata AN , Engelman JA , Faber AC . The BCL2 family: key mediators of the apoptotic response to targeted anticancer therapeutics. Cancer Discov. 2015;5(5):475‐487. 25895919 10.1158/2159-8290.CD-15-0011 PMC4727530

[9] Beroukhim R , Mermel CH , Porter D , et al. The landscape of somatic copy‐number alteration across human cancers. Nature. 2010;463(7283):899‐905. 20164920 10.1038/nature08822 PMC2826709

[10] Wu X , Luo Q , Zhao P , et al. MGMT‐activated DUB3 stabilizes MCL1 and drives chemoresistance in ovarian cancer. Proc Natl Acad Sci USA. 2019;116(8):2961‐2966. 30718431 10.1073/pnas.1814742116 PMC6386650

[11] Torres‐Adorno AM , Lee J , Kogawa T , et al. Histone Deacetylase inhibitor enhances the efficacy of MEK inhibitor through NOXA‐mediated MCL1 degradation in triple‐negative and inflammatory breast cancer. Clin Cancer Res. 2017;23(16):4780‐4792. 28465444 10.1158/1078-0432.CCR-16-2622 PMC5559319

[12] Richter‐Larrea JA , Robles EF , Fresquet V , et al. Reversion of epigenetically mediated BIM silencing overcomes chemoresistance in Burkitt lymphoma. Blood. 2010;116(14):2531‐2542. 20570860 10.1182/blood-2010-02-268003

[13] Garrison SP , Jeffers JR , Yang CY , et al. Selection against PUMA gene expression in Myc‐driven B‐cell lymphomagenesis. Mol Cell Biol. 2008;28(17):5391‐5402. 18573879 10.1128/MCB.00907-07 PMC2519737

[14] Rampino N , Yamamoto H , Ionov Y , et al. Somatic frameshift mutations in the BAX gene in colon cancers of the microsatellite mutator phenotype. Science. 1997;275(5302):967‐969. 9020077 10.1126/science.275.5302.967

[15] Singh R , Letai A , Sarosiek K . Regulation of apoptosis in health and disease: the balancing act of BCL‐2 family proteins. Nat Rev Mol Cell Bio. 2019;20(3):175‐193. 30655609 10.1038/s41580-018-0089-8 PMC7325303

[16] Chen H , Li CY , Peng XX , et al. A pan‐cancer analysis of enhancer expression in nearly 9000 patient samples. Cell. 2018;173(2):386‐399. 29625054 10.1016/j.cell.2018.03.027 PMC5890960

[17] Heinz S , Romanoski CE , Benner C , et al. The selection and function of cell type‐specific enhancers. Nat Rev Mol Cell Bio. 2015;16(3):144‐154. 25650801 10.1038/nrm3949 PMC4517609

[18] Buenrostro JD , Giresi PG , Zaba LC , et al. Transposition of native chromatin for fast and sensitive epigenomic profiling of open chromatin, DNA‐binding proteins and nucleosome position. Nat Methods. 2013;10(12):1213‐1218. 24097267 10.1038/nmeth.2688 PMC3959825

[19] Eom YH , Kim HS , Lee A , et al. BCL2 as a subtype‐specific prognostic marker for breast cancer. J Breast Cancer. 2016;19(3):252‐260. 27721874 10.4048/jbc.2016.19.3.252 PMC5053309

[20] Corces MR , Granja JM , Shams S , et al. The chromatin accessibility landscape of primary human cancers. Science. 2018;362(6413):eaav1898. 30361341 10.1126/science.aav1898 PMC6408149

[21] Llambi F , Moldoveanu T , Tait SWG , et al. A unified model of mammalian BCL‐2 protein family interactions at the mitochondria. Mol Cell. 2011;44(4):517‐531. 22036586 10.1016/j.molcel.2011.10.001 PMC3221787

[22] Leverson JD , Sampath D , Souers AJ , et al. Found in translation: how preclinical research is guiding the clinical development of the BCL2‐selective inhibitor venetoclax. Cancer Discov. 2017;7(12):1376‐1393. 29146569 10.1158/2159-8290.CD-17-0797 PMC5728441

[23] Leverson JD , Phillips DC , Mitten MJ , et al. Exploiting selective BCL‐2 family inhibitors to dissect cell survival dependencies and define improved strategies for cancer therapy. Sci Transl Med. 2015;7(279):279ra40. 10.1126/scitranslmed.aaa4642 25787766

[24] Lonsdale J , Thomas J , Salvatore M , et al. The genotype‐tissue expression (GTEx) project. Nat Genet. 2013;45(6):580‐585. 23715323 10.1038/ng.2653 PMC4010069

[25] Tang ZF , Li CW , Kang BX , et al. GEPIA: a web server for cancer and normal gene expression profiling and interactive analyses. Nucleic Acids Res. 2017;45(W1):W98‐W102. 28407145 10.1093/nar/gkx247 PMC5570223

[26] Buenrostro JD , Wu B , Chang HY , et al. ATAC‐seq: a method for assaying chromatin accessibility genome‐wide. Curr Protoc Mol Biol. 2015;109(1):21.29.1‐21.29.9. 10.1002/0471142727.mb2129s109 PMC4374986 25559105

[27] Li H , Durbin R . Fast and accurate short read alignment with Burrows‐Wheeler transform. Bioinformatics. 2009;25(14):1754‐1760. 19451168 10.1093/bioinformatics/btp324 PMC2705234

[28] Feng JX , Liu T , Qin B , et al. Identifying ChIP‐seq enrichment using MACS. Nat Protoc. 2012;7(9):1728‐1740. 22936215 10.1038/nprot.2012.101 PMC3868217

[29] Zhang Y , Liu T , Meyer CA , et al. Model‐based analysis of ChIP‐Seq (MACS). Genome Biol. 2008;9(9):R137. 18798982 10.1186/gb-2008-9-9-r137 PMC2592715

[30] Heinz S , Benner C , Spann N , et al. Simple combinations of lineage‐determining transcription factors prime cis‐regulatory elements required for macrophage and B cell identities. Mol Cell. 2010;38(4):576‐589. 20513432 10.1016/j.molcel.2010.05.004 PMC2898526

[31] Thorvaldsdóttir H , Robinson JT , Mesirov JP . Integrative Genomics Viewer (IGV): high‐performance genomics data visualization and exploration. Brief Bioinform. 2012;14(2):178‐192. 22517427 10.1093/bib/bbs017 PMC3603213

[32] Gao JJ , Aksoy BA , Dogrusoz U , et al. Integrative analysis of complex cancer genomics and clinical profiles using the cBioPortal. Sci Signal. 2013;6(269):pl1. 23550210 10.1126/scisignal.2004088 PMC4160307

[33] Heintzman ND , Stuart RK , Hon G , et al. Distinct and predictive chromatin signatures of transcriptional promoters and enhancers in the human genome. Nat Genet. 2007;39(3):311‐318. 17277777 10.1038/ng1966

[34] Heintzman ND , Hon GC , Hawkins RD , et al. Histone modifications at human enhancers reflect global cell‐type‐specific gene expression. Nature. 2009;459(7243):108‐112. 19295514 10.1038/nature07829 PMC2910248

[35] Kim TH , Barrera LO , Zheng M , et al. A high‐resolution map of active promoters in the human genome. Nature. 2005;436(7052):876‐880. 15988478 10.1038/nature03877 PMC1895599

[36] Creyghton MP , Cheng AW , Welstead GG , et al. Histone H3K27ac separates active from poised enhancers and predicts developmental state. Proc Natl Acad Sci USA. 2010;107(50):21931‐21936. 21106759 10.1073/pnas.1016071107 PMC3003124

[37] Rada‐Iglesias A , Bajpai R , Swigut T , et al. A unique chromatin signature uncovers early developmental enhancers in humans. Nature. 2011;470(7333):279‐283. 21160473 10.1038/nature09692 PMC4445674

[38] Siddiqui WA , Ahad A , Ahsan H . The mystery of BCL2 family: Bcl‐2 proteins and apoptosis: an update. Arch Toxicol. 2015;89(3):289‐317. 25618543 10.1007/s00204-014-1448-7

[39] Abed MN , Abdullah MI , Richardson A . Antagonism of Bcl‐X‐L is necessary for synergy between carboplatin and BH3 mimetics in ovarian cancer cells. J Ovarian Res. 2016;9(25):1‐9. 27080533 10.1186/s13048-016-0234-y PMC4832520

[40] Williams J , Lucas PC , Griffith KA , et al. Expression of Bcl‐xL in ovarian carcinoma is associated with chemoresistance and recurrent disease. Gynecol Oncol. 2005;96(2):287‐295. 15661210 10.1016/j.ygyno.2004.10.026

[41] Chen JM , Zhang X , Lentz C , et al. miR‐193b regulates Mcl‐1 in melanoma. Am J Pathol. 2011;179(5):2162‐2168. 21893020 10.1016/j.ajpath.2011.07.010 PMC3204027

[42] Cimmino A , Calin GA , Fabbri M , et al. miR‐15 and miR‐16 induce apoptosis by targeting BCL2. Proc Natl Acad Sci USA. 2005;102(39):13944‐13949. 16166262 10.1073/pnas.0506654102 PMC1236577

[43] Shimizu S , Takehara T , Hikita H , et al. The let‐7 family of microRNAs inhibits Bcl‐xL expression and potentiates sorafenib‐induced apoptosis in human hepatocellular carcinoma. J Hepatol. 2010;52(5):698‐704. 20347499 10.1016/j.jhep.2009.12.024

[44] Gong J , Zhang JP , Li B , et al. MicroRNA‐125b promotes apoptosis by regulating the expression of Mcl‐1, Bcl‐w and IL‐6R. Oncogene. 2013;32(25):3071‐3079. 22824797 10.1038/onc.2012.318

[45] Zhang L , Li XJ , Chao YL , et al. KLF4, a miR‐32‐5p targeted gene, promotes cisplatin‐induced apoptosis by upregulating BIK expression in prostate cancer. Cell Commun Signal. 2018;16(1):53. 30176890 10.1186/s12964-018-0270-x PMC6122640

[46] Borst A , Haferkamp S , Grimm J , et al. BIK is involved in BRAF/MEK inhibitor induced apoptosis in melanoma cell lines. Cancer Lett. 2017;404:70‐78. 28720543 10.1016/j.canlet.2017.07.005

[47] Mann J , Githaka JM , Buckland TW , et al. Non‐canonical BAD activity regulates breast cancer cell and tumor growth via 14‐3‐3 binding and mitochondrial metabolism. Oncogene. 2019;38(18):3325‐3339. 30635657 10.1038/s41388-018-0673-6 PMC6756016

[48] Sastry KSR , Al‐Muftah MA , Li P , et al. Targeting proapoptotic protein BAD inhibits survival and self‐renewal of cancer stem cells. Cell Death Differ. 2014;21(12):1936‐1949. 25215949 10.1038/cdd.2014.140 PMC4227153

[49] Bui NLC , Pandey V , Zhu T , et al. Bad phosphorylation as a target of inhibition in oncology. Cancer Lett. 2018;415:177‐186. 29175460 10.1016/j.canlet.2017.11.017

[50] Luo YW , Wang XY , Wang HR , et al. High Bak expression is associated with a favorable prognosis in breast cancer and sensitizes breast cancer cells to paclitaxel. PLoS ONE. 2015;10(9):e0138955. 26406239 10.1371/journal.pone.0138955 PMC4583467

[51] Meijerink JPP , Mensink EJBM , Wang K , et al. Hematopoietic malignancies demonstrate loss‐of‐function mutations of BAX. Blood. 1998;91(8):2991‐2997. 9531611

[52] Kratz CP , Han SS , Rosenberg PS , et al. Variants in or near KITLG, BAK1, DMRT1, and TERT‐CLPTM1L predispose to familial testicular germ cell tumour. J Med Genet. 2011;48(7):473‐476. 21617256 10.1136/jmedgenet-2011-100001 PMC3131696

[53] Slager SL , Skibola CF , Di Bernardo MC , et al. Common variation at 6p21.31 (BAK1) influences the risk of chronic lymphocytic leukemia. Blood. 2012;120(4):843‐846. 22700719 10.1182/blood-2012-03-413591 PMC3412347

[54] Dawson MA . The cancer epigenome: concepts, challenges, and therapeutic opportunities. Science. 2017;355(6330):1147‐1152. 28302822 10.1126/science.aam7304

[55] Nechiporuk T , Kurtz SE , Nikolova O , et al. The TP53 apoptotic network is a primary mediator of resistance to BCL2 inhibition in AML cells. Cancer Discov. 2019;9(7):910‐925. 31048320 10.1158/2159-8290.CD-19-0125 PMC6606338

[56] Chen X , Guo Y , Ouyang T , et al. Co‐mutation of TP53 and PIK3CA in residual disease after neoadjuvant chemotherapy is associated with poor survival in breast cancer. J Cancer Res Clin Oncol. 2019;145(5):1235‐1242. 30806788 10.1007/s00432-019-02873-8 PMC11810230

[57] Cardenas C , Montagna MK , Pitruzzello M , et al. Adipocyte microenvironment promotes Bclxl expression and confers chemoresistance in ovarian cancer cells. Apoptosis. 2017;22(4):558‐569. 28012060 10.1007/s10495-016-1339-x

[58] Green DR , Kroemer G . The pathophysiology of mitochondrial cell death. Science (New York, NY). 2004;305(5684):626‐629. 10.1126/science.1099320 15286356

[59] O'Neill KL , Huang K , Zhang JJ , et al. Inactivation of prosurvival Bcl‐2 proteins activates Bax/Bak through the outer mitochondrial membrane. Gene Dev. 2016;30(8):973‐988. 27056669 10.1101/gad.276725.115 PMC4840302

[60] Chen C‐H , Chuang S‐M , Yang M‐F , et al. A novel function of YWHAZ/β‐catenin axis in promoting epithelial‐mesenchymal transition and lung cancer metastasis. Mol Cancer Res. 2012;10(10):1319‐1331. 22912335 10.1158/1541-7786.MCR-12-0189

[61] Kambach DM , Sodi VL , Lelkes PI , et al. ErbB2, FoxM1 and 14‐3‐3ζ prime breast cancer cells for invasion in response to ionizing radiation. Oncogene. 2014;33(5):589‐598. 23318431 10.1038/onc.2012.629 PMC3966179

[62] Whale AD , Colman L , Lensun L , et al. Functional characterization of a novel somatic oncogenic mutation of PIK3CB. Signal Transduct Tar. 2017;2:17063. 10.1038/sigtrans.2017.63 PMC5740215 29279775

[63] Pridham KJ , Le L , Guo S , et al. PIK3CB/p110beta is a selective survival factor for glioblastoma. Neuro Oncol. 2018;20(4):494‐505. 29016844 10.1093/neuonc/nox181 PMC5909664

[64] Chen L , Yang L , Yao L , et al. Characterization of PIK3CA and PIK3R1 somatic mutations in Chinese breast cancer patients. Nat Commun. 2018;9(1):1357. 29636477 10.1038/s41467-018-03867-9 PMC5893593

[65] Thorpe LM , Spangle JM , Ohlson CE , et al. PI3K‐p110α mediates the oncogenic activity induced by loss of the novel tumor suppressor PI3K‐p85α. Proc Natl Acad Sci USA. 2017;114(27):7095‐7100. 28630349 10.1073/pnas.1704706114 PMC5502636

[66] Liu XJ , He YJ , Li F , et al. Caspase‐3 promotes genetic instability and carcinogenesis. Mol Cell. 2015;58(2):284‐296. 25866249 10.1016/j.molcel.2015.03.003 PMC4408780

[67] Deng L , Gao XL , Liu BJ , et al. NMT1 inhibition modulates breast cancer progression through stress‐triggered JNK pathway. Cell Death Dis. 2018;9(12):1143. 30446635 10.1038/s41419-018-1201-x PMC6240078

[68] Singh A , Singh N , Behera D , et al. Role of polymorphic XRCC6 (Ku70)/XRCC7 (DNA‐PKcs) genes towards susceptibility and prognosis of lung cancer patients undergoing platinum based doublet chemotherapy. Mol Biol Rep. 2018;45(3):253‐261. 29397516 10.1007/s11033-018-4158-z

[69] Schoenfelder S , Fraser P . Long‐range enhancer–promoter contacts in gene expression control. Nat Rev Genet. 2019;20(8):437‐455. 31086298 10.1038/s41576-019-0128-0

[70] van Steensel B , Furlong EEM . The role of transcription in shaping the spatial organization of the genome. Nat Rev Mol Cell Bio. 2019;20(6):327‐337. 30886333 10.1038/s41580-019-0114-6 PMC7116054

[71] Zheng H , Xie W . The role of 3D genome organization in development and cell differentiation. Nat Rev Mol Cell Bio. 2019;20(9):535‐550. 31197269 10.1038/s41580-019-0132-4

[72] Beagrie RA , Scialdone A , Schueler M , et al. Complex multi‐enhancer contacts captured by genome architecture mapping. Nature. 2017;543(7646):519‐524. 28273065 10.1038/nature21411 PMC5366070

