# Supplementary figures and images for: B‐cell lymphoma 2 family genes show a molecular pattern of spatiotemporal heterogeneity in gynaecologic and breast cancer
Source: Cell Prolif. 2020 May 17;53(6):e12826. doi: 10.1111/cpr.12826 (PMC7309952; doi:10.1111/cpr.12826)

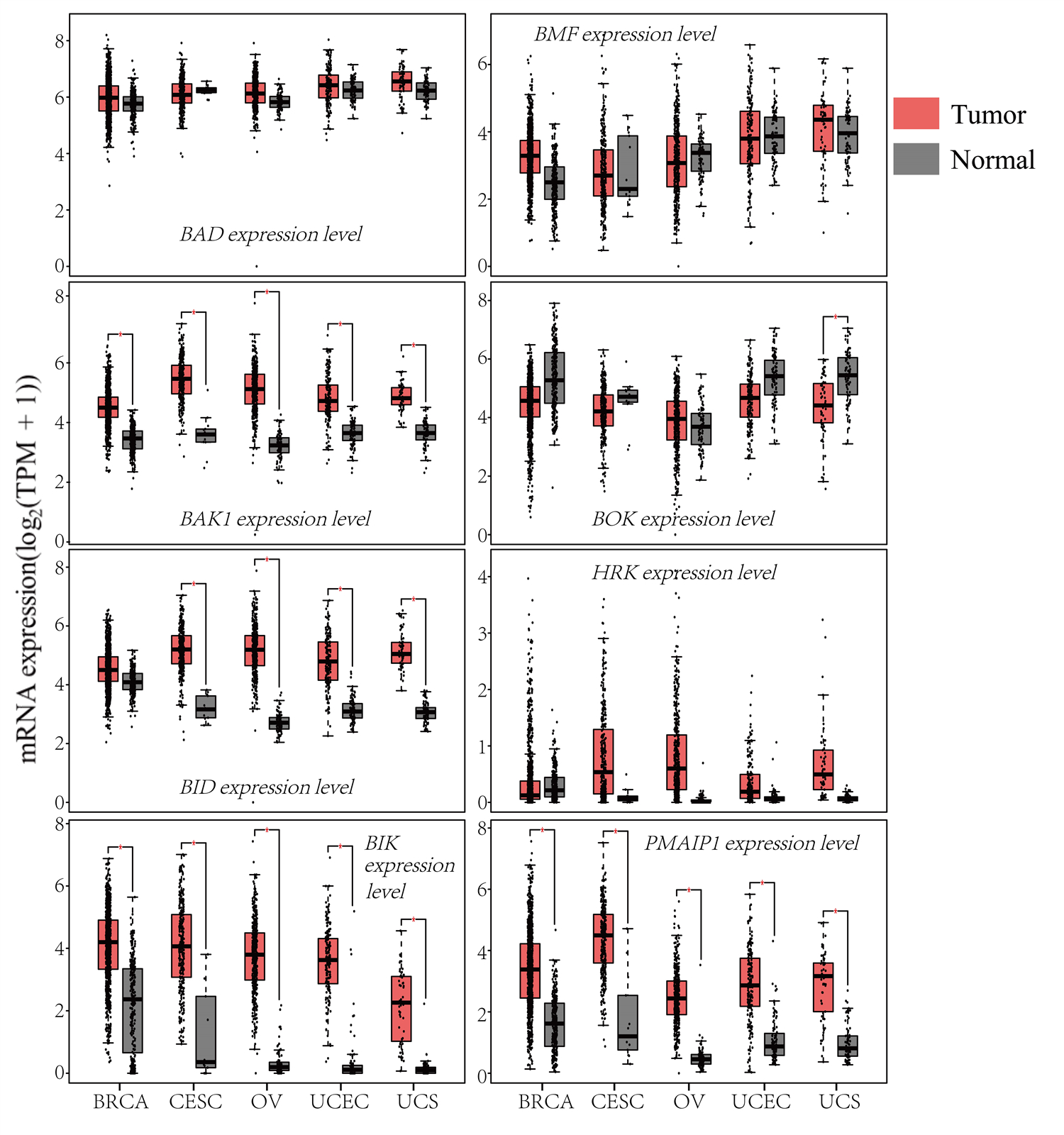

Supplement: Supplementary file 1 — Fig S1 [file CPR-53-e12826-s001.jpg]

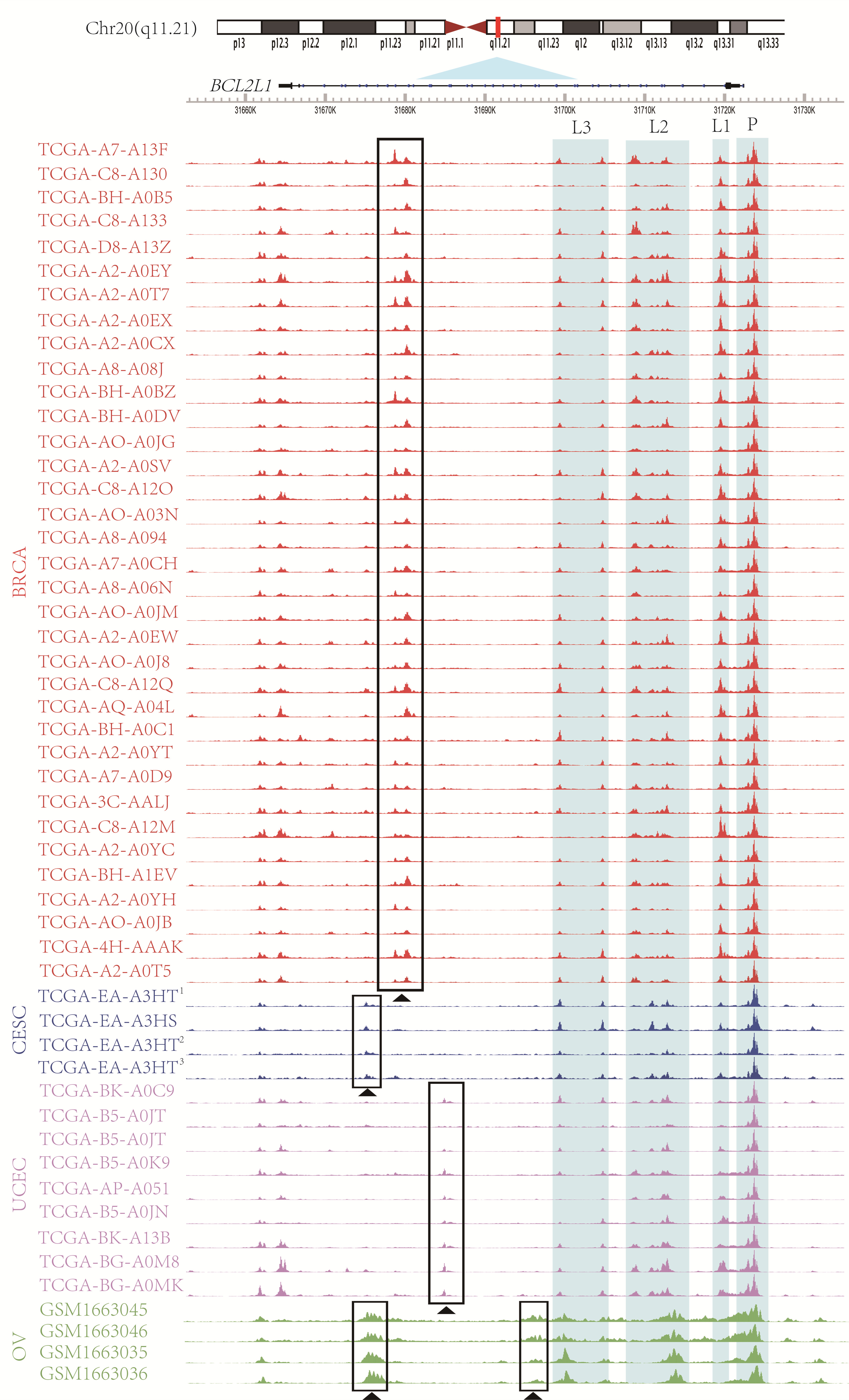

Supplement: Supplementary file 2 — Fig S2 [file CPR-53-e12826-s002.jpg]

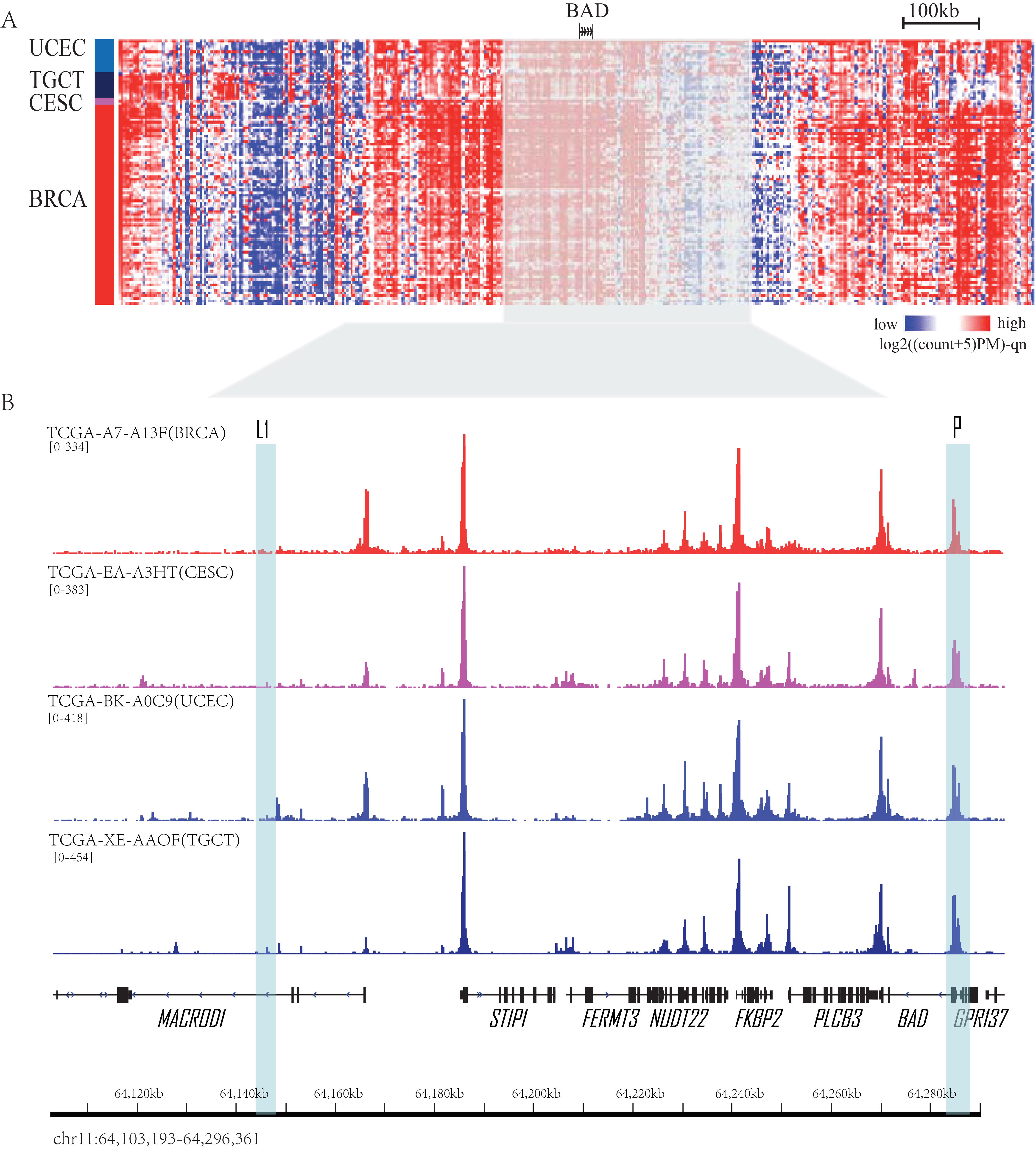

Supplement: Supplementary file 3 — Fig S3 [file CPR-53-e12826-s003.jpg]

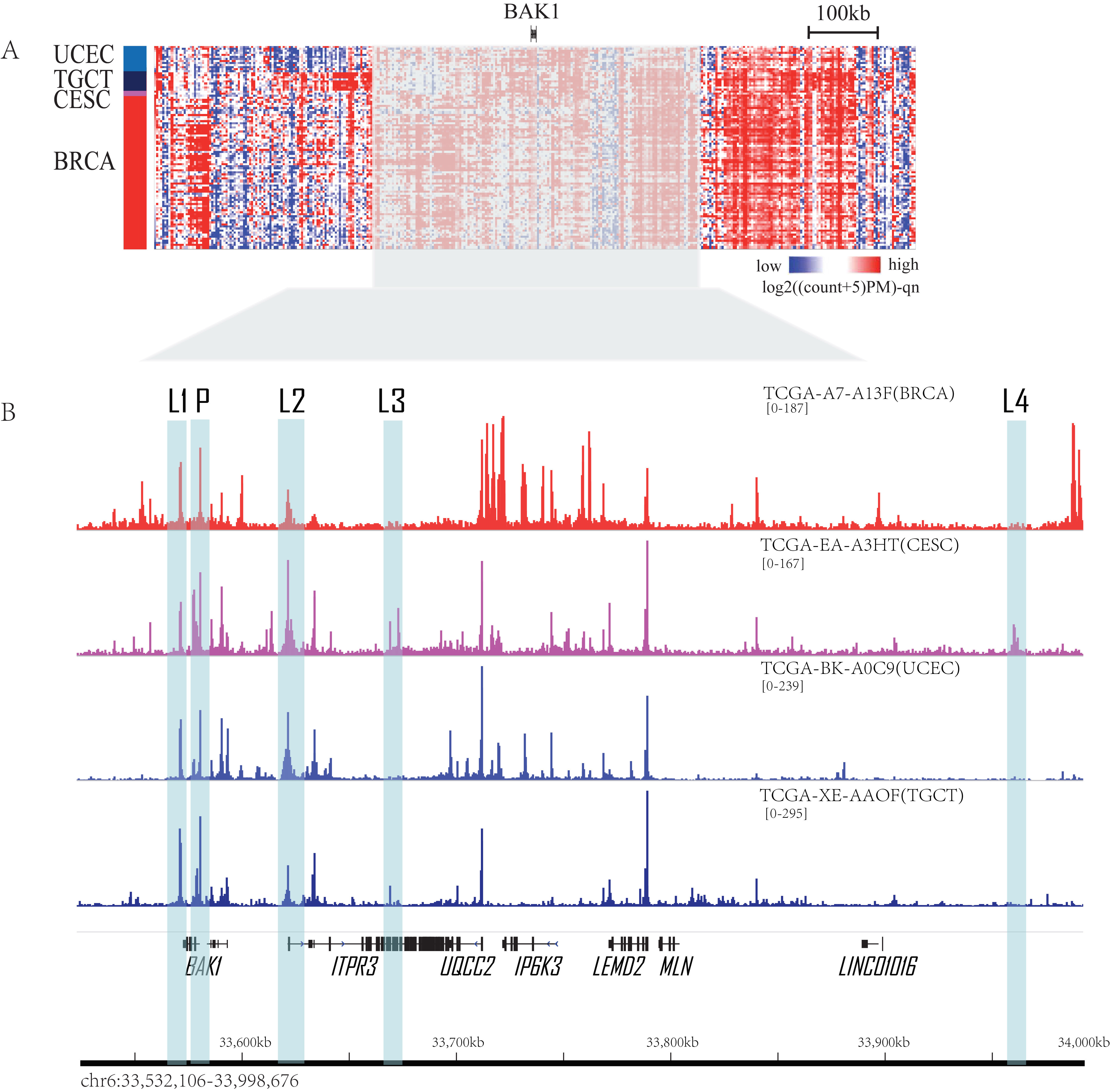

Supplement: Supplementary file 4 — Fig S4 [file CPR-53-e12826-s004.jpg]

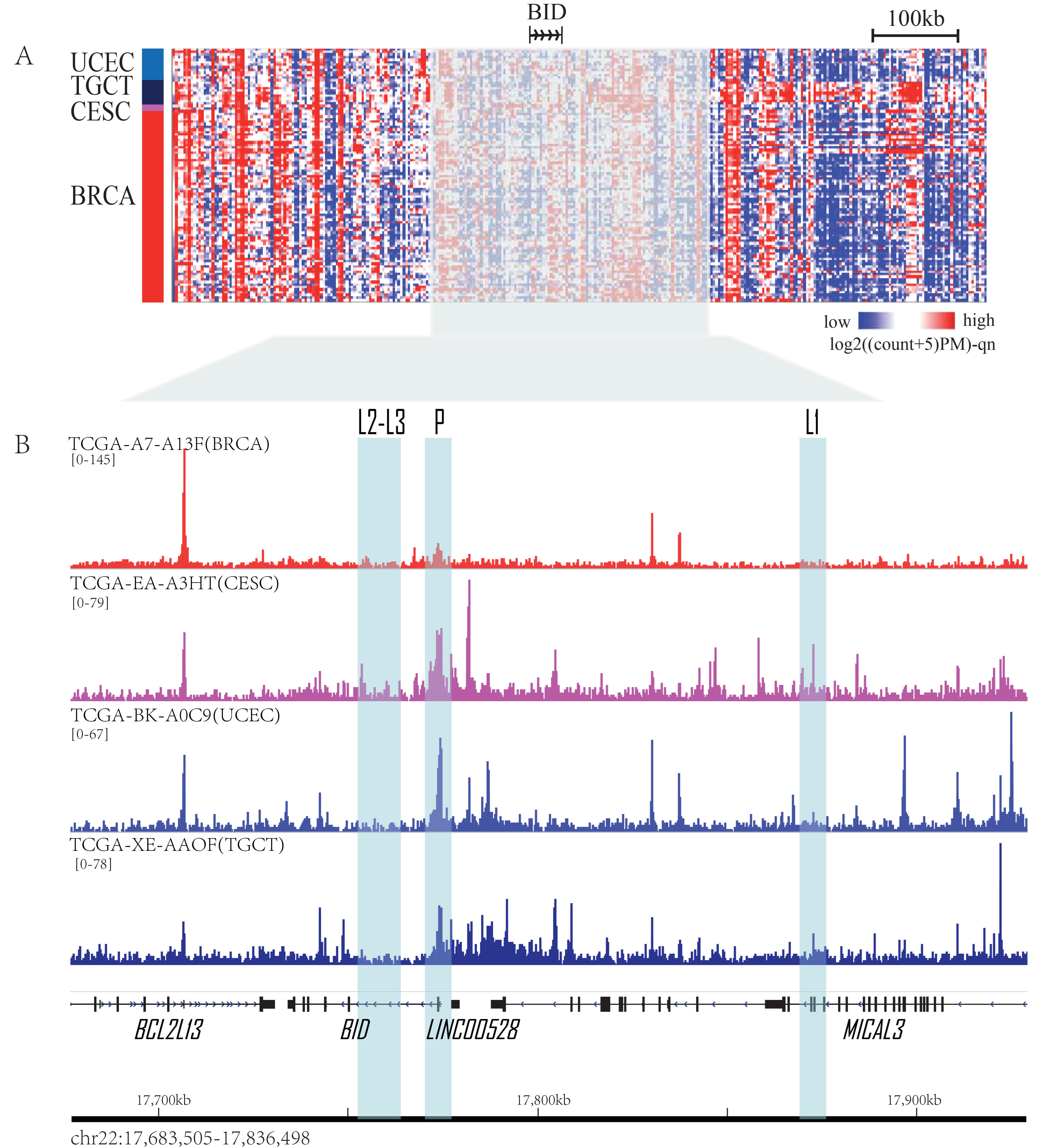

Supplement: Supplementary file 5 — Fig S5 [file CPR-53-e12826-s005.jpg]

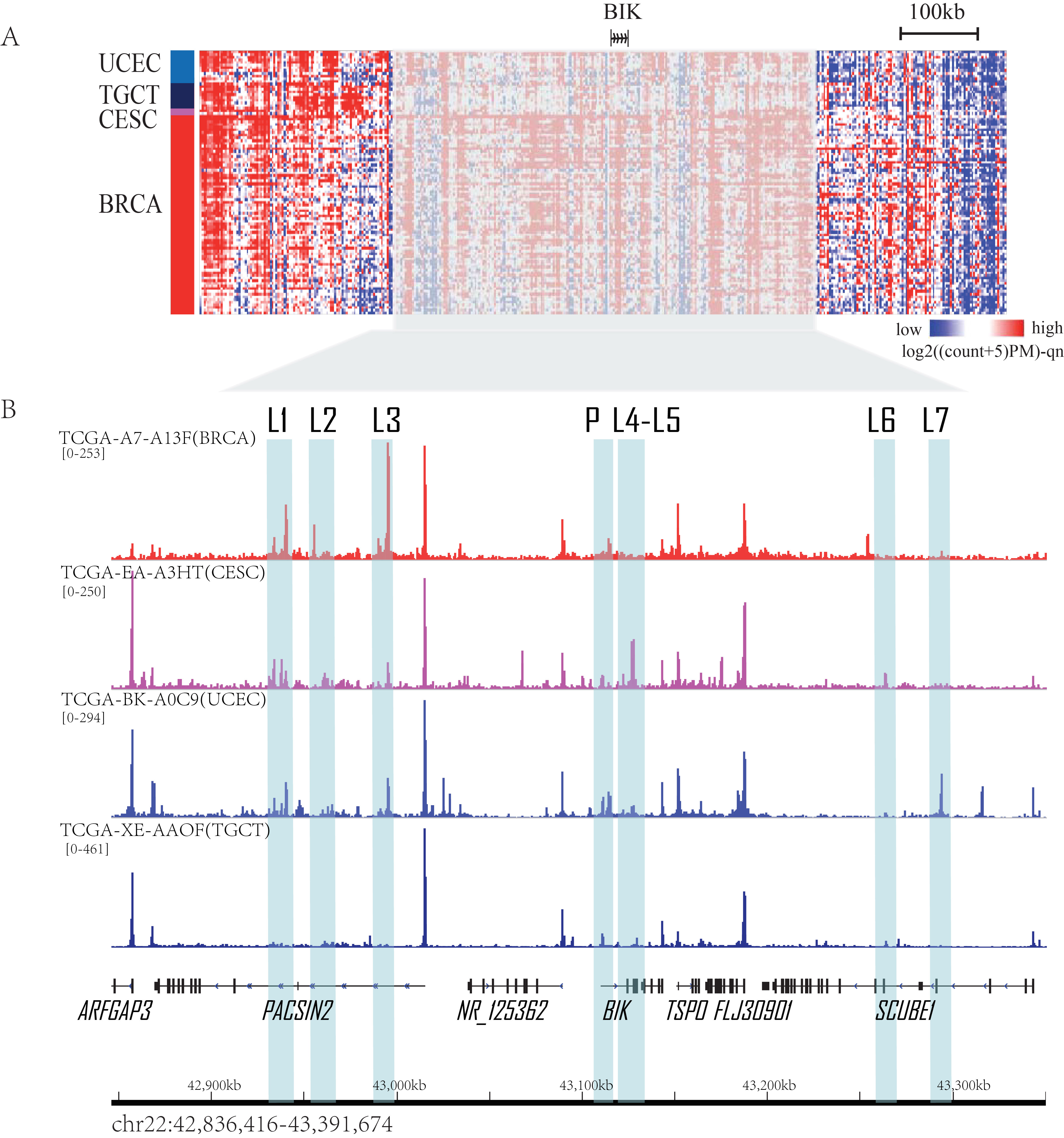

Supplement: Supplementary file 6 — Fig S6 [file CPR-53-e12826-s006.jpg]

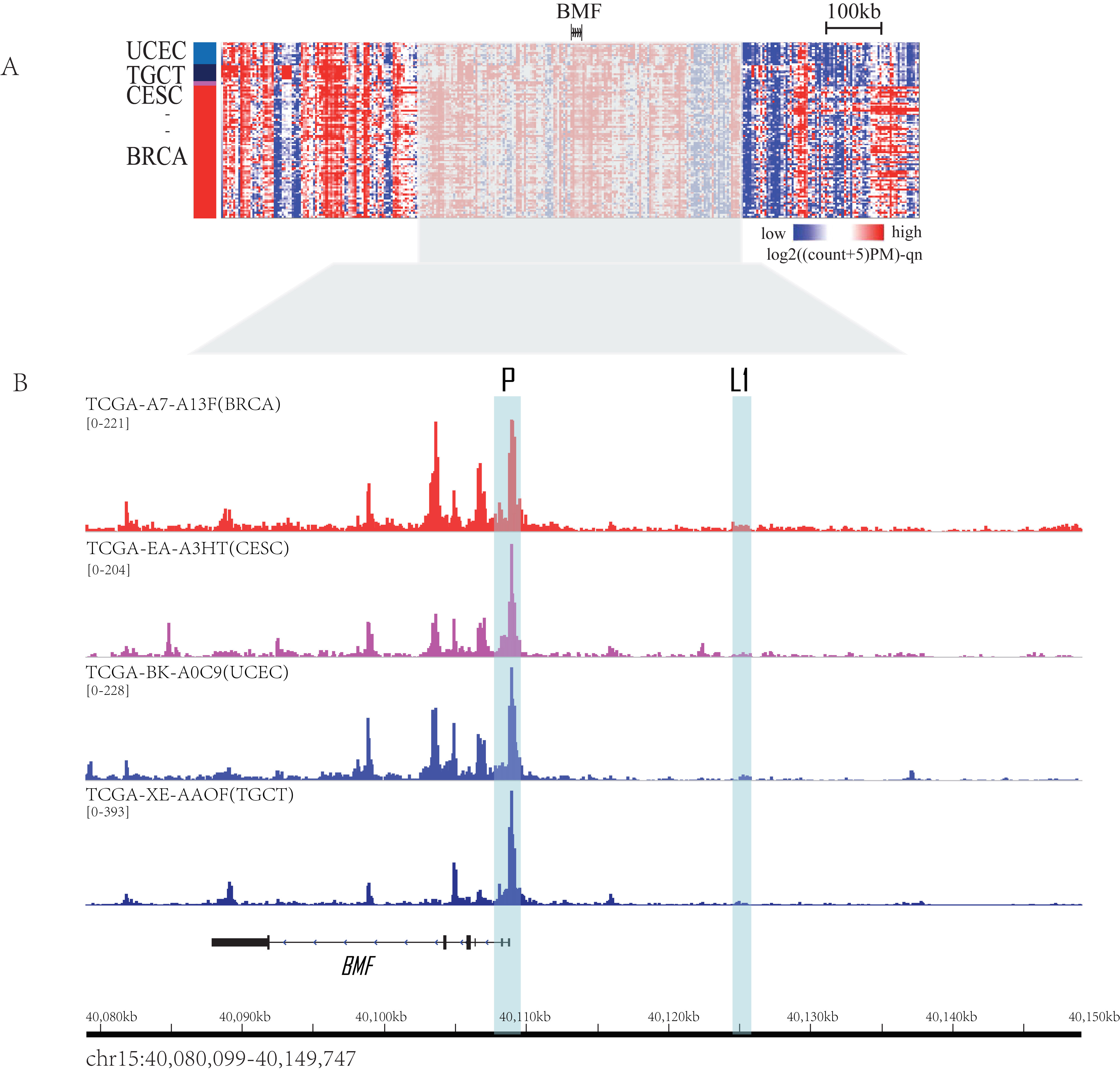

Supplement: Supplementary file 7 — Fig S7 [file CPR-53-e12826-s007.jpg]

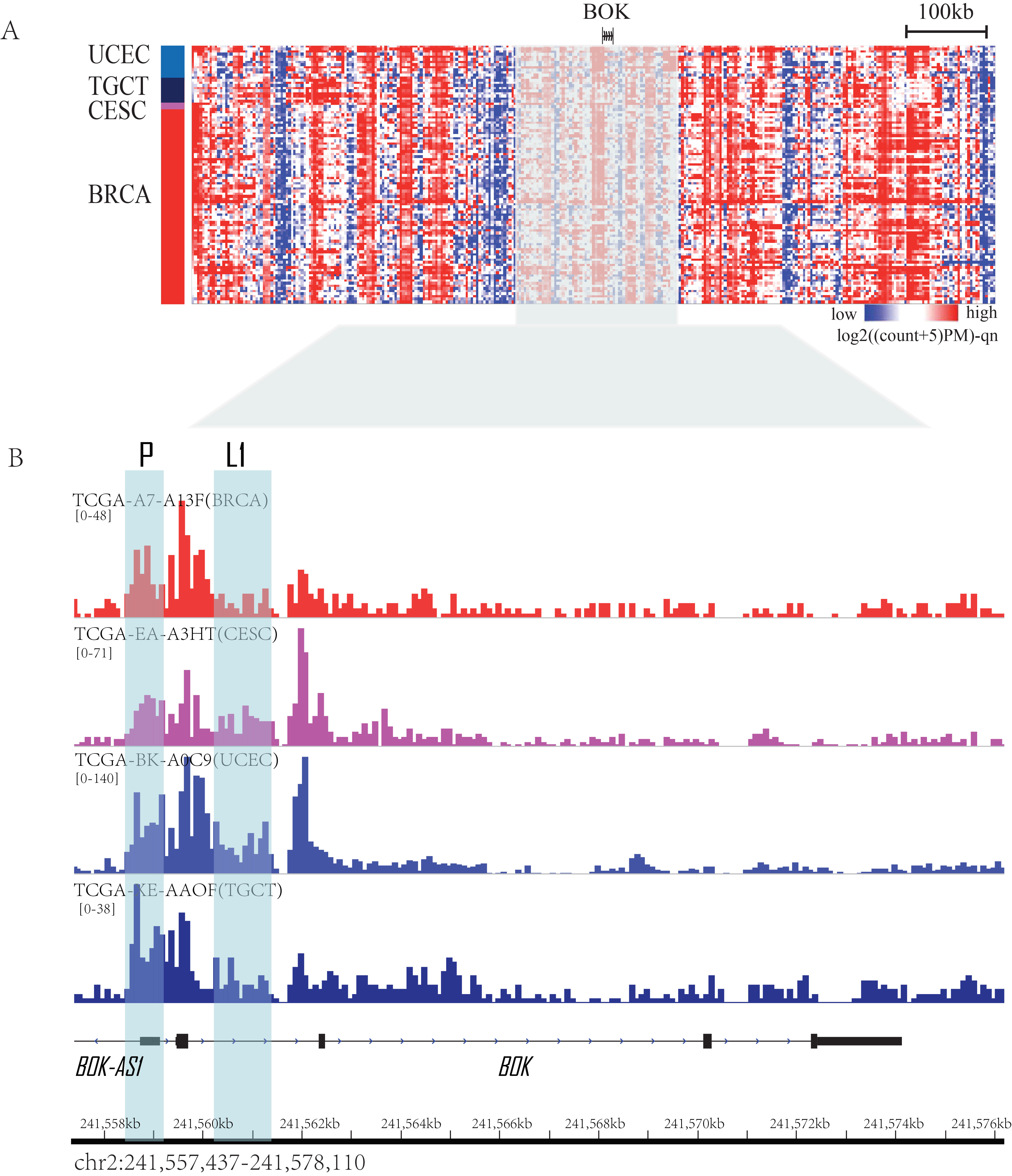

Supplement: Supplementary file 8 — Fig S8 [file CPR-53-e12826-s008.jpg]

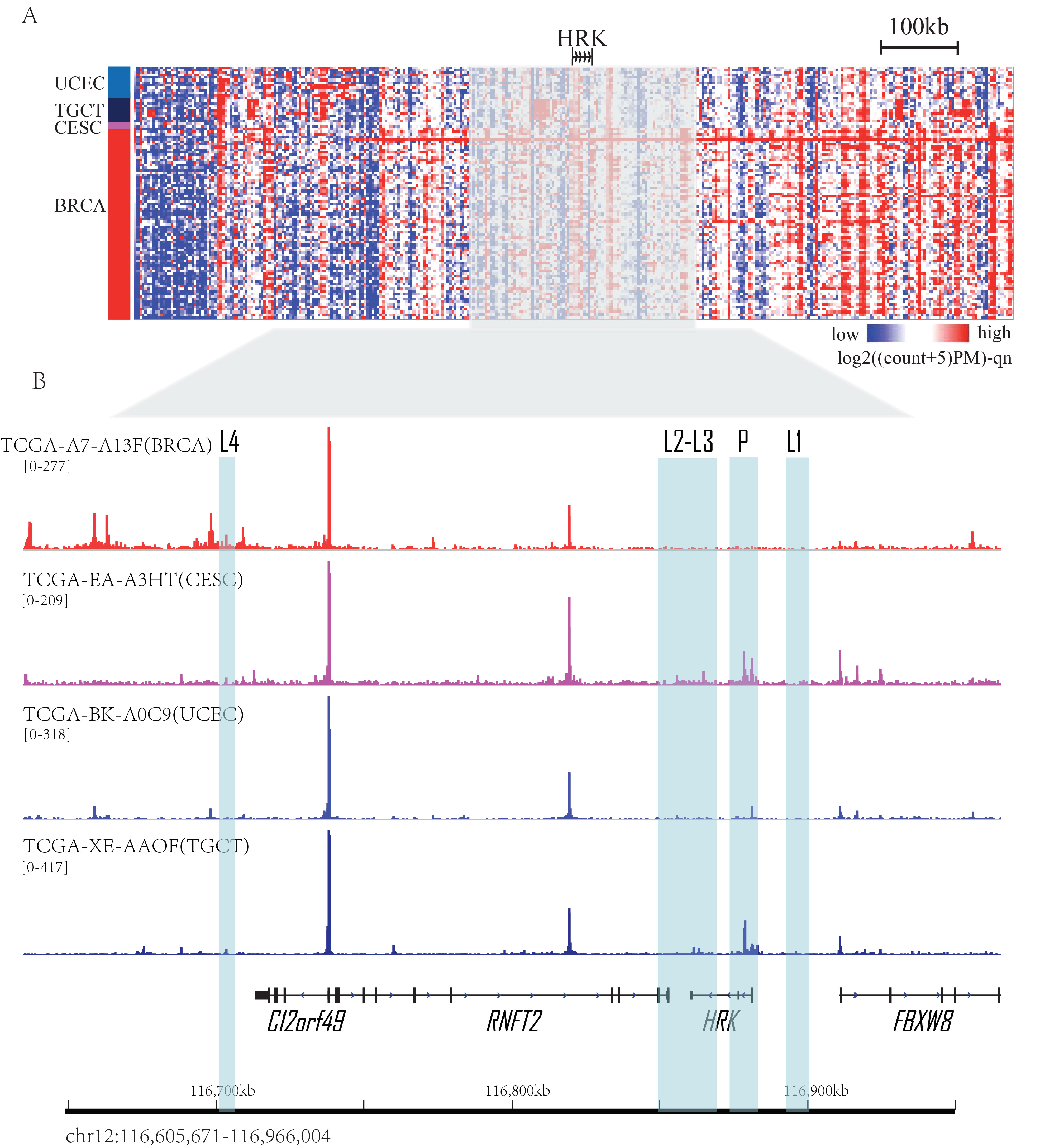

Supplement: Supplementary file 9 — Fig S9 [file CPR-53-e12826-s009.jpg]

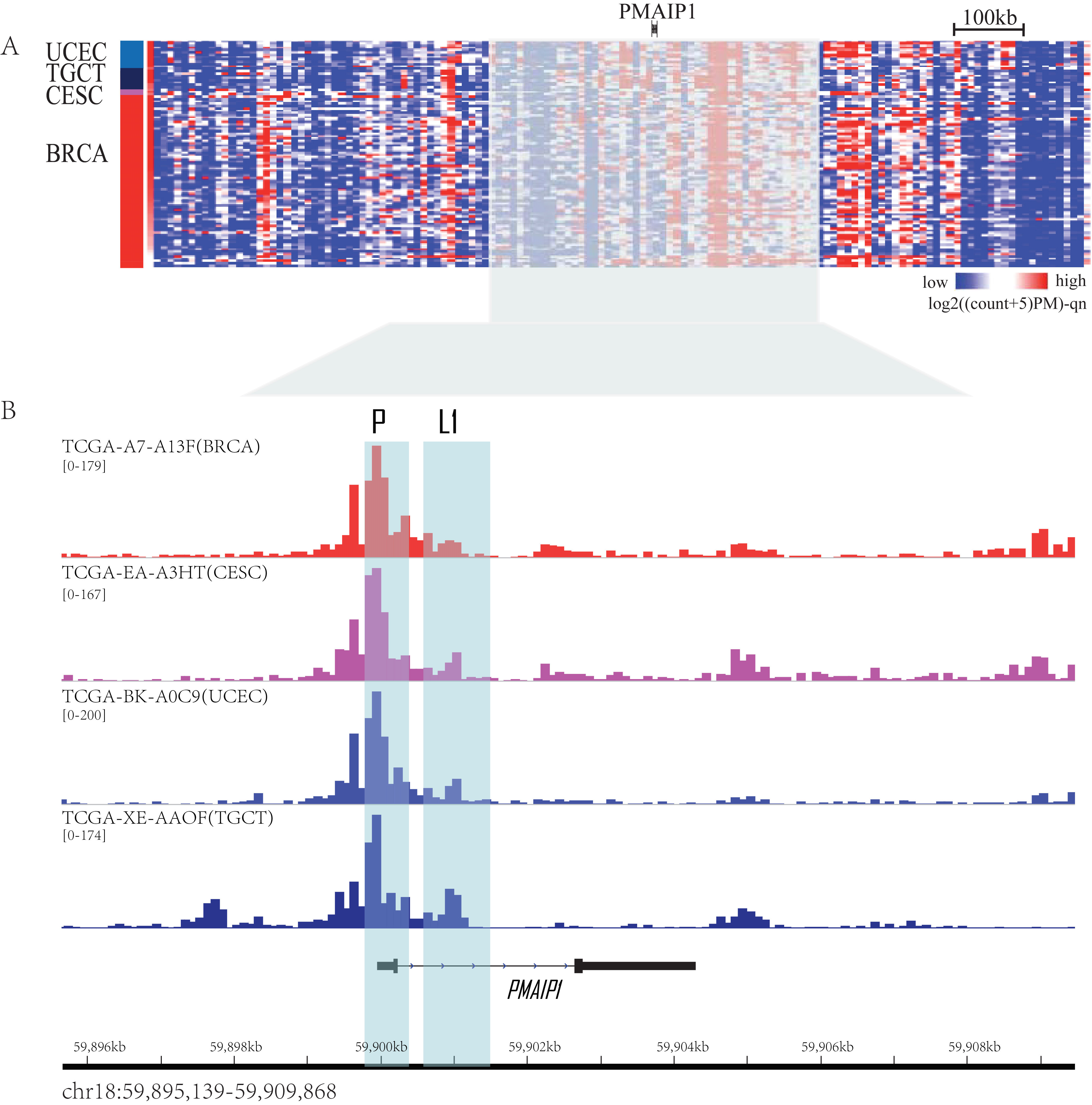

Supplement: Supplementary file 10 — Fig S10 [file CPR-53-e12826-s010.jpg]
