# Supplementary material for: B‐cell lymphoma 2 family genes show a molecular pattern of spatiotemporal heterogeneity in gynaecologic and breast cancer
Source: Cell Prolif. 2020 May 17;53(6):e12826. doi: 10.1111/cpr.12826 (PMC7309952; doi:10.1111/cpr.12826)
Supplement: Supplementary file 11 — Table S1‐S3 [file CPR-53-e12826-s011.docx]

**Supporting information**

**Supplementary Figures Legends:**

**Supplemental Figure 1. The mRNA expression patterns for additional pro/anti-apoptotic BCL2 family members in gynecologic cancer.** Up-regulated mRNA expression levels of *BAD, BAK1, BID, BIK, BMF, BOK, HRK,* and *PMAIP1* in gynecologic and breast cancer compared with normal tissue. The red boxes represent tumor samples, while the black boxes represent normal samples. The red star indicates statistical significance (P<0.01).

**Supplementary Figure 2. Chromatin accessibility of the *BCL2L1* gene locus across genomic locations in gynecologic and breast cancer.** Normalized open chromatin tracks of the *BCL2L1* gene locus in gynecologic and breast cancer (including BRCA (N= 35), CESC (N=4), UCEC (N=9) and OV (N=4)). Three inferred peaks at promoter regions are indicated (L1 to L3, positioned at chr20: 31,713,198-31,714,227, chr20: 31,716,244-31,717,493, and chr20: 31,720,069-31,720,570). Highly variable distal regulatory landscapes are marked by black triangles at chr20:31,652,584 to 31,734, or 966, depending on the tumor type.

**Supplementary Figure 3. The long-range gene regulation of the *BAD* gene locus with variable levels of expression in gynecologic and breast cancer. (A)** Heatmap representation of the open chromatin region around the *BAD* gene locus within 500 kb. Each row represents a patient sample (*N* = 74 for BRCA, *N*= 2 for CESC, *N* = 11 for UCEC, *N* = 9 for TGCT), while the range of the column represents various genomic regions. Red color represents normalized accessibility, and blue color indicates closed chromatin regions. **(B)** Normalized open chromatin tracks of the *BAD* gene locus in four samples. The inferred promoter peaks are marked by bright blue bands (L1, positioned at chr11: 64,141,241-64,141,742). The region shown ranges from chr11:64,103,193 to 64,296,361.

**Supplementary Figure 4. The long-range gene regulation of the *BAK1* gene locus with variable levels of expression in gynecologic and breast cancer. (A)** Heatmap representation of the open chromatin region around the *BAK1* gene locus within 500 kb. Each row represents a patient sample (*N* = 74 for BRCA, *N*= 2 for CESC, *N* = 11 for UCEC, *N* = 9 for TGCT), while the range of the column represents various genomic regions. Red color represents normalized accessibility, and blue color indicates closed chromatin regions. **(B)** Normalized open chromatin tracks of the *BAK1* gene locus in four samples. Four inferred promoter peaks are marked by bright blue bands (L1 to L4, positioned at chr6: 33,623,781-33,624,282, chr6: 33,623,781-33,624,282, chr6: 33,668,892-33,669,393, and chr6: 33,950,408-33,950,909, respectively). The region shown ranges from chr6:33,532,106 to 33,998,676.

**Supplementary Figure 5. The long-range gene regulation of the *BID* gene locus with variable levels of expression in gynecologic and breast cancer. (A)** Heatmap representation of the open chromatin region around the *BID* gene locus within 500 kb. Each row represents a patient sample (*N* = 74 for BRCA, *N*= 2 for CESC, *N* = 11 for UCEC, *N* = 9 for TGCT); the range of the column represents various genomic regions. Red color represents normalized accessibility, blue color indicates closed chromatin region. **(B)** Normalized open chromatin tracks of the *BID* gene locus in four samples. Three inferred promoter peaks are marked by bright blue bands (L1 to L3, positioned at chr22: 17,872,448-17,872,949, chr22: 17,773,195-17,773,696, and chr22: 17,770,550-17,771,051, respectively). The region shown ranges from chr22: 17,683,505 to 17,836,498.

**Supplementary Figure 6. The long-range gene regulation of the *BIK* gene locus with variable levels of expression in gynecologic and breast cancer. (A)** Heatmap representation of the open chromatin region around the *BIK* gene locus within 500 kb. Each row represents a patient sample (*N* = 74 for BRCA, *N*= 2 for CESC, *N* = 11 for UCEC, *N* = 9 for TGCT); the range of the column represents various genomic regions. Red color represents normalized accessibility, blue color indicates closed chromatin region. **(B)** Normalized open chromatin tracks of the *BIK* gene locus in four samples. Seven inferred promoter peaks are marked by bright blue bands (L1 to L7, peaks positioned at chr22: 42,940,163-42,940,664, chr22: 42,955,197-42,955,698, chr22: 42,994,296-42,995,688, chr22: 43,111,120-43,111,621, chr22: 43,113,865-43,115,380, chr22: 43,278,158-43,278,659, and chr22: 43,293,169-43,294,185, respectively). The region shown ranges from chr22: 42,836,416 to 43,391,674.

**Supplementary Figure 7. The long-range gene regulation of the *BMF* gene locus with variable levels of expression in gynecologic and breast cancer. (A)** Heatmap representation of the open chromatin region around the *BMF* gene locus within 500 kb. Each row represents a patient sample (*N* = 74 for BRCA, *N*= 2 for CESC, *N* = 11 for UCEC, *N* = 9 for TGCT), while the range of the column represents various genomic regions. Red color represents normalized accessibility, blue color indicates closed chromatin region. **(B)** Normalized open chromatin tracks of the *BMF* gene locus in four samples. The inferred promoter are marked by bright blue bands (L1, positioned at chr15: 40,124,951-40,125,452). The region shown ranges from chr15:40,080,099 to 40,149,747.

**Supplementary Figure 8. The long-range gene regulation of the *BOK* gene locus with variable levels of expression in gynecologic and breast cancer. (A)** Heatmap representation of the open chromatin region around the *BOK* gene locus within 500 kb. Each row represents a patient sample (*N* = 74 for BRCA, *N*= 2 for CESC, *N* = 11 for UCEC, *N* = 9 for TGCT), while the range of the column represents various genomic regions. Red color represents normalized accessibility, blue color indicates closed chromatin region. **(B)** Normalized open chromatin tracks of the *BOK* gene locus in four samples. The inferred promoter peaks are marked by bright blue bands (L1, positioned at chr2: 241,560,358-241,561,512). The region shown ranges from chr2: 241,557,437 to 241,578,110.

**Supplementary Figure 9. The long-range gene regulation of the *HRK* gene locus with variable levels of expression in gynecologic and breast cancer. (A)** Heatmap representation of the open chromatin region around the *HRK* gene locus within 500 kb. Each row represents a patient sample (*N* = 74 for BRCA, *N*= 2 for CESC, *N* = 11 for UCEC, *N* = 9 for TGCT), while the range of the column represents various genomic regions. Red color represents normalized accessibility, blue color indicates closed chromatin region. **(B)** Normalized open chromatin tracks of the *HRK* gene locus in four samples. Four inferred promoter peaks are marked by bright blue bands (L1 to L4, positioned at chr12: 116,882,672-116,883,173, chr12: 116,858,516-116,859,017, chr12: 116,876,397-116,876,898, chr12: 116,717,743-116,718,244, respectively). The region shown ranges from chr12: 116,605,671 to 116,966,004.

**Supplementary Figure10. The long-range gene regulation of the *PMAIP1* gene locus with variable levels of expression in gynecologic and breast cancer. (A)** Heatmap representation of the open chromatin region around the *PMAIP1* gene locus within 500 kb. Each row represents a patient sample (*N* = 74 for BRCA, *N*= 2 for CESC, *N* = 11 for UCEC, *N* = 9 for TGCT), while the range of the column represents various genomic regions. Red color represents normalized accessibility, blue color indicates closed chromatin region. **(B)** Normalized open chromatin tracks of the *PMAIP1* gene locus in four samples. The inferred promoter peaks are marked by bright blue bands (L1, positioned at chr18: 59,900,723-59,902,526). The region shown ranges from chr18: 59,895,139 to 59,909,868.

**Supplementary Tables**

Supplementary Table 1. Sources of published histone modification ChIP-seq datasets

Supplementary Table 2. Nomenclature of BCL2 family members expressed in gynecologic and breast cancers

Supplementary Table 3. Alteration frequencies of BCL2 family genes in patients with pan-cancer

**Supplementary Table 1. Sources of published histone modification ChIP-seq datasets.**

| **Tissue** | **Dataset** | **Accession number** | **Reference** |
| --- | --- | --- | --- |
| Breast | H3K4me1 ChIP-seq | GEO: GSM986083 | Theodorou V, et al. GATA3 acts upstream of FOXA1 in mediating ESR1 binding by shaping enhancer accessibility. Genome Res. 2013 PMID: 23172872 |
| Breast | H3K27ac ChIP-seq | GEO: GSM2476262 | Liu Y, et al. Transcriptional landscape of the human cell cycle. Proc. Natl. Acad. Sci. U.S.A. 2017 PMID: 28289232 |
| Cervix | H3K27ac ChIP-seq | GEO: GSM2990418 | Shi L, et al. Histone H3.3 G34 Mutations Alter Histone H3K36 and H3K27 Methylation In Cis. J. Mol. Biol. 2018 PMID: 29689253 |
| Cervix | H3K27ac ChIP-seq | GEO: GSM733684 | ENCODE Project Consortium , et al. An integrated encyclopedia of DNA elements in the human genome. Nature 2012 PMID: 22955616 |
| Endometrium | EP300 ChIP-seq | GEO: GSM1010759 | Gertz J, et al. Distinct properties of cell-type-specific and shared transcription factor binding sites. Mol. Cell 2013 PMID: 24076218 |
| Endometrium | TEAD4 ChIP-seq | GEO: GSM1010885 | Gertz J, et al. Distinct properties of cell-type-specific and shared transcription factor binding sites. Mol. Cell 2013 PMID: 24076218 |
| Ovary | H3K27ac ChIP-seq | GEO: GSM1663045 | Coetzee SG, et al. Cell-type-specific enrichment of risk-associated regulatory elements at ovarian cancer susceptibility loci. Hum. Mol. Genet. 2015 PMID: 25804953 |
| Ovary | H3K27ac ChIP-seq | GEO: GSM1663046 | Coetzee SG, et al. Cell-type-specific enrichment of risk-associated regulatory elements at ovarian cancer susceptibility loci. Hum. Mol. Genet. 2015 PMID: 25804953 |
| Ovary | H3K27ac ChIP-seq | GEO: GSM1663035 | Coetzee SG, et al. Cell-type-specific enrichment of risk-associated regulatory elements at ovarian cancer susceptibility loci. Hum. Mol. Genet. 2015 PMID: 25804953 |
| Breast, Cervix, Endometrium | ATAC-seq |  | Corces, M. R. et al. The chromatin accessibility landscape of primary human cancers. Science 2018 PMID: 30361341 |

**Supplementary Table 2. Nomenclature of BCL2 family members expressed in gynecologic and breast cancers**

| **Name** | **Alternative name** |
| --- | --- |
| BCL2 | Bcl-2, PPP1R50 |
| BCL2L1 | BCL-XL/S, BCL2L, BCLX, BCLXL, BCLXS, Bcl-X, PPP1R52 |
| BCL2L2 | BCL-W, BCLW, PPP1R51 |
| MCL1 | BCL2L3, EAT, MCL1-ES, MCL1L, MCL1S, Mcl-1, TM, bcl2-L-3 |
| BAX | BCL2L4 |
| BAK1 | BCL2L7, BAK-LIKE, CDN1 |
| BAD | BCL2L8, BBC2 |
| BOK | BCL2L9, BOKL |
| BCL2L11 | BAM, BIM, BOD |
| BMF | BMF |
| BID | FP497 |
| PMAIP1 | APR, NOXA |
| HRK | DP5, HARAKIRI |
| BBC3 | PUMA, JFY-1, JFY1 |
| BIK | BIP1, BP4, NBK |

**Supplementary Table 3. Alteration frequencies of BCL2 family genes in patients with pan-cancer**

| **Alteration** | **UCS** | **UCEC** | **OV** | **BRCA** | **CESC** |
| --- | --- | --- | --- | --- | --- |
| **Gene alteration** | 47.37%(57 cases) | 27.22%(529 cases) | 28.89%(585 cases) | 20.2%(1084 cases) | 17.51%(297 cases) |
| **Amplification** | 35.09%(20 cases) | 11.91%(63 cases) | 18.46%(108 cases) | 15.04%(163 cases) | 9.76%(29 cases) |
| **Deletion** | 3.51%(2 cases) | 2.46%(13 cases) | 7.52%(44cases) | 2.68%(29 cases) | 3.37%(10 cases) |
| **Mutation** | 5.26%(3 cases) | 11.34%(60 cases) | 1.37%(8 cases) | 1.85%(20 cases) | 3.03%(9 cases) |
